# Regulating microglial miR-155 transcriptional phenotype alleviates Alzheimer’s-induced retinal vasculopathy by limiting Clec7a/Galectin-3^+^ neurodegenerative microglia

**DOI:** 10.1186/s40478-022-01439-z

**Published:** 2022-09-08

**Authors:** Haoshen Shi, Zhuoran Yin, Yosef Koronyo, Dieu-Trang Fuchs, Julia Sheyn, Miyah R. Davis, Jered W. Wilson, Milica A. Margeta, Kristen M. Pitts, Shawn Herron, Seiko Ikezu, Tsuneya Ikezu, Stuart L. Graham, Vivek K. Gupta, Keith L. Black, Mehdi Mirzaei, Oleg Butovsky, Maya Koronyo-Hamaoui

**Affiliations:** 1 Department of Neurosurgery, Maxine Dunitz Neurosurgical Research Institute, Cedars-Sinai Medical Center, 127 S. San Vicente Blvd., Los Angeles, CA A6212 USA; 2 Department of Neurology, Brigham and Women’s Hospital, Harvard Medical School, Boston, MA USA; 3 Department of Ophthalmology, Massachusetts Eye and Ear Infirmary, Harvard Medical School, Boston, MA USA; 4 Department of Pharmacology and Experimental Therapeutics, Boston University School of Medicine, Boston, MA USA; 5 Department of Neuroscience, Mayo Clinic Florida, Jacksonville, FL USA; 6 Department of Health and Human Sciences, Macquarie University, Sydney, NSW Australia; 7 Department of Molecular Sciences, Macquarie University, Sydney, NSW Australia; 8 Department of Clinical Medicine, Department of Molecular Sciences and Australian Proteome Analysis Facility, Macquarie University, Sydney, NSW Australia; 9 Evergrande Center for Immunologic Diseases, Brigham and Women’s Hospital, Harvard Medical School, Boston, MA USA; 10 Department of Biomedical Sciences, Division of Applied Cell Biology and Physiology, Cedars-Sinai Medical Center, Los Angeles, CA USA

**Keywords:** Microglia, microRNA, Inflammation, Vascular damage, Retinopathy, Alzheimer’s disease

## Abstract

Single cell RNA sequencing studies identified novel neurodegeneration-associated microglial (MGnD/DAM) subtypes activated around cerebral amyloid plaques. Micro-RNA (miR)-155 of the TREM2-APOE pathway was shown to be a key transcriptional regulator of MGnD microglial phenotype. Despite growing interest in studying manifestations of Alzheimer’s disease (AD) in the retina, a CNS organ accessible to noninvasive high-resolution imaging, to date MGnD microglia have not been studied in the AD retina. Here, we discovered the presence and increased populations of Clec7a^+^ and Galectin-3^+^ MGnD microglia in retinas of transgenic APP_SWE_/PS1_L166P_ AD-model mice. Conditionally targeting MGnD microglia by miR-155 ablation via the tamoxifen-inducible Cre^ERT2^ system in APP_SWE_/PS1_L166P_ mice diminished retinal Clec7a^+^ and Galectin-3^+^ microglial populations while increasing homeostatic P2ry12^+^ microglia. Retinal MGnD microglia were often adhering to microvessels; their depletion protected the inner blood-retina barrier and reduced vascular amyloidosis. Microglial miR-155 depletion further limits retinal inflammation. Mass spectrometry analysis revealed enhanced retinal PI3K-Akt signaling and predicted IL-8 and Spp1 decreases in mice with microglia-specific miR-155 knockout. Overall, this study identified MGnD microglia in APP_SWE_/PS1_L166P_ mouse retina. Transcriptional regulation of these dysfunctional microglia mitigated retinal inflammation and vasculopathy. The protective effects of microglial miR-155 ablation should shed light on potential treatments for retinal inflammation and vascular damage during AD and other ocular diseases.

**Supplementary Information:**

The online version contains supplementary material available at 10.1186/s40478-022-01439-z.

## Introduction

Persistent, low-grade inflammation is a key feature of Alzheimer’s disease (AD) neuropathology. Previous histological, proteomic, and single-cell RNA sequencing (RNAseq) analyses, as well as epidemiologic and genome-wide association studies (GWAS), revealed that innate immune processes are central to AD pathogenesis [12, 34, 40, 42, 50]. Augmented neuroinflammation in AD has been associated with detrimental microglial and astrocytic phenotypes [15, 22, 25, 35, 37, 43, 47, 65]. Microglia are resident innate immune cells of the central nervous system (CNS); these phagocytic cells play important roles in fighting infection, removing misfolded proteins and cell debris, mediating inflammatory responses, and promoting synaptic remodeling and tissue regeneration [51]. In the healthy brain, the homeostatic (M0) microglia population has been defined, with a phenotype regulated by the transforming growth factor beta (TGFβ) signaling pathway [11, 35]. Upon activation by pathogens or damage signals, microglia undergo morphological and transcriptional changes that may lead to secretion of molecules causing neuronal damage [41]. A recent RNAseq analysis identified a distinct neurodegeneration and disease-associated microglial phenotype (MGnD/DAM) that is regulated via the triggering receptor expressed on the myeloid cells 2-apolipoprotein E (TREM2-APOE) pathway and is closely associated with cerebral amyloid β-protein (Aβ) plaques, a pathological hallmark of AD [43, 47]. The study identified microRNA-155 (miR-155) as a post-translational regulator of this microglial phenotype switch from M0 to MGnD.

MiR-155 is a small multifunctional, single-stranded non-coding RNA molecule that plays important roles in hematopoiesis, inflammation, immunity, vasculopathy and cancer [14, 60, 61, 92]. Earlier studies demonstrated that miR-155 directly targets rapamycin-insensitive companion of mammalian target of rapamycin (RICTOR) and SMAD family member 1 (SMAD-1), and can regulate various signaling pathways such as the phosphoinositide 3-kinase-protein kinase B (PI3K-Akt), vascular remodeling and growth, tight junctions (TJs) and blood–brain barrier (BBB) integrity [59, 67, 91]. Targeting miR-155 by genetic ablation and anti-miR-155 treatment in the SOD1-G93A (SOD1) mouse model restored the population of neuroprotective M0 homeostatic microglia and enhanced their phagocytic capability in the CNS [11].

The retina is a CNS extension of the brain that is readily accessible to live imaging at high specificity and sensitivity. Numerous reports in the past decade have described the manifestation of pathological changes specific to AD in the neurosensory retina of animal models and AD patients, including amyloidosis, tauopathy, neurodegeneration, vasculopathy, and inflammation [2, 3, 7, 17, 19–21, 26, 27, 29–33, 36, 44–46, 48, 54, 63, 64, 68–70, 75, 76, 79, 82, 88, 89, 96]. A recent study in postmortem retinas from AD patients revealed the presence of reactive astrocytes and microglial activation that were associated with Aβ deposition [30]. In retinal inflammatory diseases, such as age-related macular degeneration, diabetic retinopathy, and retinitis pigmentosa, retinal microglia are consistently activated and polarized to multiple reactive phenotypes [72, 73]. So far, it is unknown which phenotype of microglia is associated with AD in the retina. Moreover, the recently defined neurodegeneration-associated microglial phenotype has never been investigated in the AD-afflicted retina.

Here, we provide the first evidence for the existence of MGnD microglia in the retinas of murine models of AD. To examine the effects of targeting miR-155 on MGnD versus homeostatic microglial phenotype expression, we crossed double transgenic APP_SWE_/PS1_L166P_ mouse models of AD (herein, APP/PS1 mice) with miR-155^ fl/fl^ and Cx3cr1^CreERT2^ mice to achieve tamoxifen-inducible miR-155 knock-out specifically in the microglia. Conditional miR-155 deletion in microglia led to diminished retinal Clec7a^+^ and Galectin-3^+^ microglial subtypes as well as significantly downregulated retinal inflammation. Proteome analysis showed that targeting miR-155 in retinal microglia is associated with enhanced PI3K-Akt signaling, inhibited expression of C-X-C motif chemokine ligand-8 (Cxcl8) and secretion of phosphoprotein 1 (Spp1; also known as osteopontin or Opn). Importantly, suppressing MGnD phenotype expression by targeting microglial miR-155 protected APP/PS1 mice retina from AD-induced tight-junction degeneration and vascular amyloidosis. Our results reveal that the neurodegeneration-, amyloid- and inflammation-associated MGnD microglial phenotype plays important roles in retinal vascular dysfunction as well. Targeting transcriptional regulation of MGnD by miR-155 ablation may provide a novel treatment option for combatting retinal inflammation and protecting retinal blood vessels.

## Materials and methods

### Mice

Three mouse genotypes were used to generate create the Cx3cr1^CreERT2^-miR-155^fl/fl^-APP/PS1 (APP/PS1:miR-155cKO) mouse model with rapid Aβ deposition and microglial miR-155 conditional knockout (cKO). B6.129P2(Cg)-Cx3cr1^tm2.1(cre/ERT)Litt^/WganJ (Cx3cr1^CreERT2^) [66] and C57BL/6-Mir-155^tm1.1Ggard^/J (Mir155^fl/fl^) mice [38] were purchased from Jackson Laboratories; APP_SWE_/PS1_L166P_ (APP/PS1) mice [71] were kindly provided by Dr. Mathias Jucker (University of Tubingen). The miR-155^ fl/fl^ mice were bred with both APP/PS1 mice and Cx3cr1^CreERT2^ mice to generate the Cx3cr1^CreERT2^-miR-155^fl/fl^, and miR-155^fl/fl^-APP/PS1 genotypes. These two strains were further crossed with each other to create APP/PS1:miR-155cKO mice. Cx3cr1^WT^-miR-155fl^/fl^-APP/PS1 mice serve as controls to the APP/PS1:miR-155cKO mice. This mouse genotype specifically expresses tamoxifen-inducible Cre (Cre^ERT2^) in microglia, a method that has been validated by a previous microglial BDNF knock-out study [47, 66]. Non-Tg wild type (WT) mice were crossed with miR-155^fl/fl^ mice and Cx3cr1^CreERT2^ mice using the same methods as specified above to create Cx3cr1^CreERT2^-miR155^fl/fl^-WT (WT:miR-155cKO) and miR-155^fl/fl^-WT (WT) mice. Two consecutive doses of tamoxifen (150 mg/kg) were injected peritoneally when the animals were 6 weeks of age to introduce miR-155cKO specifically to microglia. The Institutional Animal Care and Use Committee (IACUC) at Harvard Medical School, Brigham and Women’s Hospital approved all experimental procedures involving the animals.

Two cohorts (n = 48 each) of mice were generated for protein analysis or histological examination. For each cohort, both eyes were collected from 4-month-old and from 8-month-old mice of all four genotypes described above: APP/PS1:miR-155cKO, APP/PS1, WT:miR-155cKO, and WT. Six mice were assigned to each type of analysis and each age/genotype group. For protein analyses, left and right retinas of each mouse were pooled and sonicated in RIPA lysis buffer (Thermo Fisher Scientific, #89,900) with proteinase inhibitor and phosphatase inhibitor (Thermo Fisher Scientific, #78,440). The protein concentration of each sample was determined using the Thermo Scientific Pierce BCA Protein Assay Kit (Thermo Fisher Scientific, #23,225). For histological analyses, whole right eyes were preserved in 4% paraformaldehyde (PFA) with 30% sucrose at 4 °C before whole eye cryo-section, while retinas from the left eyes were removed by orbital dissection, fixed in 4% PFA overnight, and then preserved in 1 × PBS for further analysis of isolated retinal vasculature or retinal flat mounts.

### Quantitative reverse transcription polymerase chain reaction (qRT-PCR) analyses for miR-155 expression in isolated microglia from mouse brains

Mice were euthanized in a CO_2_ chamber and then transcardially perfused with cold Hanks’ Balanced Salt Solution (HBSS). Whole brains were removed, and the left hemispheres were homogenized to form a single cell suspension, then resuspended and centrifuged in a 37/70% Percoll Plus (GE Healthcare, #17-5445-02) gradient at 800G, 23 °C, for 25 min with an acceleration of 3 and a deceleration of 1. Mononuclear cells were taken from the interface layer. The cells were stained with rat anti-Fcrls mab (1:200, clone 4G11, 3 mg/ml) [11, 47] followed by secondary detection with APC goat anti-rat IgG (1:300, Biolegend, clone Poly4054), anti-mouse CD11b-PeCy7 (1:300, eBioscience, 50-154-54), and anti-mouse Ly-6C-PerCP/Cy5.5 (1:300, Biolegend, 123,012). After staining, Ly-6C^–^CD11b^+^Fcrls^+^ cells were washed and sorted using BD FACSAria^™^ II (BD Bioscience) or MoFlo astrios.

Total RNA including miRNA was extracted using RNeasy Plus Micro Kit (Qiagen, #74034) according to the manufacturer’s protocol for purification of total RNA containing small RNAs from cells. For qRT-PCR analyses, total RNA with specific miRNA probes (Applied Biosystems) were used after reverse transcription reaction (high-capacity cDNA Reverse Transcription Kit; Applied Biosystems, #4368814). All miRNA amplifications were performed with commercially available FAM-labeled Taqman probes (Mmu-miR-155, Assay ID:002571, Thermo Fisher Scientific, #4427975). Real-time PCR reaction was performed using QuantStudio^™^ 7 (Applied Biosystems). The data of miRNAs were presented as relative expression normalized to U6 (U6 snRNA, Assay ID: 001973, Thermo Fisher Scientific, #4427975) as mean ± standard errors of the means (SEMs).

### Retinal flat mount and immunofluorescence staining

The PFA-fixed retinas were first blocked in tris-buffered saline (TBS) containing 0.3% Triton X-100 and 10% normal donkey serum for 1 h at room temperature (RT). Following this, the retinas were incubated in various combinations of primary antibodies in blocking buffers for 2 days at 4 °C. Primary antibody combinations included Clec7a/TMEM119/lectin, Galectin-3/TMEM119/lectin and Spp1/Iba1/Gfap (see antibody details and dilutions in Table 1). Retinas were then washed extensively in TBS containing 0.3% Triton X-100 for 24 h, followed by incubation of secondary antibodies (see antibody details and dilutions in Table 1). Retinas were finally washed extensively in TBS for 24 h before being mounted on slides using ProLong Gold antifade reagent with DAPI (Invitrogen #P36935). Representative Z-stack images were repeatedly captured at the same tissue thickness by using a Carl Zeiss 780 confocal microscope or an Axio Imager Z1 fluorescence microscope (Carl Zeiss MicroImaging, Inc.) equipped with ApoTome, AxioCam MRm, and AxioCam HRc cameras. All qualitative observations on retinal flat mounts were performed by screening the whole retinal tissue of each mouse.

**Table 1:** List of antibodies

Antibody or reagent	Source species	Dilution	Application	Source	Catalog. #
*Primary antibody*					
Fcrls mAb	Rat	1:200	FCM	Butovsky Lab	N/A
CD11b-PeCy7	Rat	1:300	FCM	eBioscience	50-154-54
Ly-6C-PerCP/Cy5.5	Rat	1:300	FCM	BioLegend	123,012
Clec7a mAb	Rat	1:100 1:50	IF-CS IF-FL	InvivoGen	Mabg-mdect
TMEM119 mAb	Rabbit	1:500 1:200	IF-CS IF-FL	Synaptic Systems	400,011
Alexa Fluor 488-conjugated tomato lectin	Lycopersicon esculentum	1:200 1:100 1:100	IF-CS IF-FL IF-RMVI	DyLight	DL-1174
Galectin-3 pAb	Goat	1:100 1:50 1:1000	IF-CS IF-FL WB	R&D Systems	AF1197
IBA1	Rabbit	1:100 1:50	IF-CS IF-FL	Fujifilm Wako Chemicals	019-19,741
IBA1	Goat	1:100 1:50 1:1000	IF-CS IF-FL WB	Novus Biologicals	NB100-1028
Aβ_42_ (12F4) mAb	Mouse	1:200	IF-CS	BioLegend	805,501
P2ry12	Rabbit	1:200 1:500	IF-CS WB	Butovsky Lab	N/A
APOE pAb	Goat	1:200	IF-CS	Millipore Sigma	AB947
phospho-NF-κB p65 Ser536 mAb	Rabbit	1:1000	WB	Cell Signaling Technology	3033S
NF-κB p65 mAb	Rabbit	1:1000	WB	Cell Signaling Technology	8242S
TNF-α mAb	Rabbit	1:1000	WB	Cell Signaling Technology	11948S
IL-1β mAb	Mouse	1:1000	WB	Cell Signaling Technology	12242S
EIF3c pAb	Rabbit	1:1000	WB	Cell Signaling Technology	2068S
NDUFA6 pAb	Rabbit	1:1000	WB	Thermo Fisher Scientific	PA5-97,259
NDUFA10 pAb	Rabbit	1:1000	WB	Thermo Fisher Scientific	PA5-21,474
Spp1	Goat	1:100 1:20 1:500	IF-CS IF-FL WB	R&D Systems	AF808
GFAP	Rat	1:50	IF-FL	Invitrogen	13–0300
4G8 mAb	Mouse	1:200	IF- RMVI	Biolegend	800,701
Aβ_40_ (11A50-B10) mAb	Mouse	1:200	IF- RMVI	Biolegend	805,401
Zonula Occluden-1 pAb	Rabbit	1:50	IF- RMVI	Thermo Fisher Scientific	61–7300
Claudin-1 pAb	Rabbit	1:75 1:1000	IF- RMVI WB	Thermo Fisher Scientific	51–9000
MMP-9	Goat	1:800	WB	R&D Systems	AF909
*Secondary antibody*					
APC anti-rat IgG clone Poly4054	Goat	1:300	FCM	BioLegend	
Cy2 (anti-Rabbit, anti-goat, anti-rat)	Donkey	1:200	IF	Jackson ImmunoResearch Laboratories	
Cy3 (anti-rabbit, anti-goat, anti-rat)	Donkey	1:200	IF	Jackson ImmunoResearch Laboratories	
Cy5 (anti-mouse, anti-rabbit, anti-rat, anti-goat)	Donkey	1:200	IF	Jackson ImmunoResearch Laboratories	
Peroxidase Donkey Anti-Rabbit IgG (H + L)	Donkey	1:10,000	WB	Jackson ImmunoResearch Laboratories	
Peroxidase Donkey Anti-Mouse IgG (H + L)	Donkey	1:10,000	WB	Jackson ImmunoResearch Laboratories	
Peroxidase Donkey Anti-Goat IgG (H + L)	Donkey	1:10,000	WB	Jackson ImmunoResearch Laboratories	

*FCM* Flow cytometry, *IF* immunofluorescence, *CS* cross-section, *FL* flat mount, *RMVI* retinal microvascular isolation, *WB* western blot, *pAb* polyclonal antibody, *mAb* monoclonal antibody, *N/A* not applicable

### Retinal cross-sections and immunofluorescence staining

Whole eye sagittal cross-sections, 14 μm thick, were prepared using a Leica cryostat system (Leica Biosystems). The cross-sections were prepared throughout dorsal to ventral and central to peripheral retina. For immunostaining, the cross-sections were first incubated in permeabilization solution (PBS + 0.25% Triton-X + 0.05% NaN_3_) for 5 min at RT, followed by blocking in 5% normal donkey serum in 1X PBS for 1 h at RT. Then the samples were incubated at 4 °C overnight in various primary antibody combinations including Clec7a/Iba1, Galectin-3/Iba1/12F4, Tmem119/12F4, P2ry12/12F4, Apoe/Iba1/12F4 and Spp1/Tmem119 (see antibody details and dilutions in Table 1). The cross-sections were then washed with 1X PBS three times, followed by incubation with secondary antibodies (see antibody details and dilutions in Table 1). The samples were finally washed with 1X PBS three times and mounted using ProLong Gold antifade reagent with DAPI (Invitrogen #P36935). Representative images were captured using a Carl Zeiss 780 confocal microscope or an Axio Imager Z1 fluorescence microscope; images for microglial counting and stereological analysis were obtained using an Axio Imager Z1 fluorescence microscope.

### Western blotting analysis

Equal amounts of total proteins were loaded onto 4–20% Tris–glycine gels (Invitrogen, #XP04205BOX) and transferred to polyvinylidene fluoride (PVDF) membranes. All membranes were blocked in Tris-buffered saline with Tween 20 (TBST; 10 mmol/L Tris–HCl buffer, pH 8.0, 150 mmol/L NaCl, and 0.1% Tween 20) with 5% bovine serum albumin (BSA) at RT for 1 h, followed by overnight incubation with primary antibodies (see antibody details and dilutions in Table 1). The membranes were then washed in TBST four times before secondary antibody incubations for 1 h, 30 min at RT (see antibody details and dilutions in Table 1). After final washing in TBST four times, the proteins were exposed using a chemiluminescence substrate kit (Thermo Fisher Scientific, #34,580), and images were obtained using the iBright imaging system (iBright imaging system; Thermo Fisher Scientific). Image Studio Lite software version 5.2 (LI-COR Biosciences, Lincoln, NE) was used to analyze protein expression. One representative blot is shown for each molecule.

### Meso scale discovery (MSD) analysis

The V-PLEX Proinflammatory Panel 1 Mouse Kit (Meso Scale Diagnostics, LLC, #K15048D) was used to examine protein expression of various inflammatory cytokines in retinal lysates. All experiments were conducted by strictly following instructions in the product manual.

### Proteome analysis by mass spectrometry

#### Sample preparation for mass spectrometry analysis

Extracted proteins were reduced using 5 mM dithiothreitol and alkylated using 10 mM iodoacetamide. The proteins (150 µg) were digested overnight using trypsin (Promega, Madison, WI). Resulting peptides were then acidified with 1% trifluoroacetic acid and purified using styrene divinylbenzene‐reverse phase sulfonate (SDB-RPS; Empore) stage tips.

#### Tandem mass tag (TMT) Labelling

Four independent 10 plex TMT experiments were carried out to accommodate the biological replicates on the conditions. Briefly, dried peptides from each sample were resuspended in 100 mM HEPES (pH 8.2) buffer and peptide concentrations were measured using the MicroBCA protein assay kit. Peptides (60 µg) from each sample were subjected to TMT labelling with 0.8 mg reagent per tube. Labelling was conducted at RT for 1 h with continuous vortexing. To quench any remaining TMT reagent and reverse the tyrosine labelling, 5% hydroxylamine (8 μl) was added to each tube followed by vortexing and incubation for 15 min at RT. For each of the respective ten plex experiments, the ten labelled samples were combined and dried down by vacuum centrifugation. Prior to High-pH reversed-phase fractionation, the digested and TMT-labelled peptide samples were cleaned using a reverse-phase C18 clean-up column (Sep-pak, Waters) and dried in a vacuum centrifuge. The peptide mixture was resuspended in loading buffer (5 mM ammonia solution, pH 10.5) and separated into a total of 96 fractions using an Agilent 1260 HPLC system equipped with a quaternary pump, degasser, and multi-wavelength detector (MWD) (set at 210-, 214- and 280-nm wavelengths). Peptides were separated on a 55-min linear gradient of 3–30% acetonitrile in 5-mM ammonia solution (pH 10.5) at a flow rate of 0.3 mL/min on an Agilent 300 Extend C18 column (3.5 μm particles, 2.1 mm inner diameter and 150 mm length). The 96 fractions were finally consolidated into eight fractions.

#### Liquid chromatography electrospray ionization tandem mass spectrometry (LC–ESI–MS/MS) data acquisition

Mass spectrometric data were collected on an Orbitrap Lumos mass spectrometer coupled to a Proxeon NanoLC-1200 UHPLC. The 100-µm capillary column was packed with 35 cm Accucore 150 resin (2.6 μm, 150 Å; Thermo Fisher Scientific). The scan sequence began with an MS1 spectrum (Orbitrap analysis, resolution 60,000, 400 − 1600 Th, automatic gain control (AGC) target 4 × 105, maximum injection time 50 ms). Data were acquired for 90 min per fraction. Analysis at the MS2 stage consisted of higher energy collision-induced dissociation (HCD), Orbitrap analysis with a resolution of 50,000, AGC of 1.25 × 10^5^, normalized collision energy (NCE) of 37, maximum injection time of 120 ms, and an isolation window at 0.5 Th. For data acquisition including field asymmetric ion mobility spectrometry (FAIMS), the dispersion voltage (DV) was set at 5000 V; the compensation voltages (CVs) were set at − 40 V, − 60 V, and − 80 V; and the TopSpeed parameter was set at 1.5 s per CV.

#### Database searching, peptide quantification, and statistical analysis

Spectra were converted to mzXML via MSconvert. Database searching included all entries from the Human UniProt Database (downloaded August 2019). The database was concatenated with one composed of all protein sequences for that database in the reverse order. Searches were performed using a 50-ppm precursor ion tolerance for total protein level profiling. The product ion tolerance was set to 0.2 Da. We selected these wide mass tolerance windows to maximize sensitivity in conjunction with Comet searches and linear discriminant analysis. TMT tags on lysine residues and peptide N-termini (+ 229.163 Da for TMT), and carbamidomethylation of cysteine residues (+ 57.021 Da) were set as static modifications, while oxidation of methionine residues (+ 15.995 Da) was set as a variable modification. Peptide-spectrum matches (PSMs) were adjusted to a 1% false discovery rate (FDR). PSM filtering was performed using a linear discriminant analysis, as described previously, and then assembled further to a final protein level FDR of 1%, using the Picked FDR method [23]. An isolation purity of at least 0.7 (70%) in the MS1 isolation window was used for samples. For each protein, the filtered peptide TMT SN values were summed to create protein quantifications. To control for different total protein loading within a TMT experiment, the summed protein quantities of each channel were adjusted to be equal within the experiment. Proteins were quantified by summing reporter ion counts across all matching PSMs, as described elsewhere [62]. Reporter ion intensities were adjusted to correct for the isotopic impurities of the different TMT reagents according to manufacturer specifications. Finally, each protein abundance measurement was scaled, such that the summed signal-to-noise for that protein across all channels equalled 100, thereby generating a relative abundance (RA) measurement. Two criteria were applied to determine significantly regulated proteins: fold change equal to or greater than 1.2 and a *P*-value equal to or less than 0.05.

#### Functional network and computational analysis

Detectable protein hierarchies, displayed as heatmaps, and a principal component analysis (PCA) were created using ClustVis (https://biit.cs.ut.ee/clustvis/). Volcano plots were created using Prism 9. A pie chart of protein annotation through evolutionary relationship (PANTHER) protein classification analysis was created using the PANTHER Classification System (http://pantherdb.org/geneListAnalysis.do). Data were analyzed using the Qiagen Ingenuity Pathway Analysis (IPA; https://digitalinsights.qiagen.com). Differentially expressed genes (with corresponding fold changes and *P*-values) were incorporated in canonical pathways and upstream regulator analyses, and were used to generate diagrams.

### Retinal vascular isolation and immunofluorescence staining

The trypsin-induced retinal digestion and vascular network isolation technique was originally developed in 1993 and subsequently modified by replacing trypsin with commercially available elastase [90]. Our modified protocol has been previously described [78, 80]. Briefly, retinas fixed in PFA were first washed in running distilled water overnight and then digested in 40 U/mL elastase solution (Merck Millipore, #324,682) for 2 h at 37 °C. After their initial digestion, the tissues were incubated in activation solution (Tris buffer at pH 8.5) overnight for extensive digestion. The next day, the retinas were transferred to Superfrost microscope slides with 1X PBS, then carefully cleaned with a rat whisker tool under a dissecting microscope to remove nonvascular tissues. This was followed by 3 washes with 1X PBS. Samples of isolated retinal vasculature were then mounted on slides carefully without prior dehydration for immunofluorescence staining, followed by incubation in blocking buffer (Dako #X0909) for 1 h at RT. Tissues were stained overnight at 4 °C with the following primary antibody combinations: claudin-1/4G8/lectin and ZO-1/11A50-B10/lectin (see antibody details and dilutions in Table 1). Tissues were then washed three times with PBS and incubated with secondary antibodies for 2 h at RT. The tissues were again washed with PBS three times and then mounted using ProLong Gold antifade reagent with DAPI (Invitrogen #P36935). Representative images were captured using a Carl Zeiss 780 confocal microscope; images for the stereological analysis were obtained using an Axio Imager Z1 fluorescence microscope.

### Microglial counting and stereological quantification

**Fig. 1:**
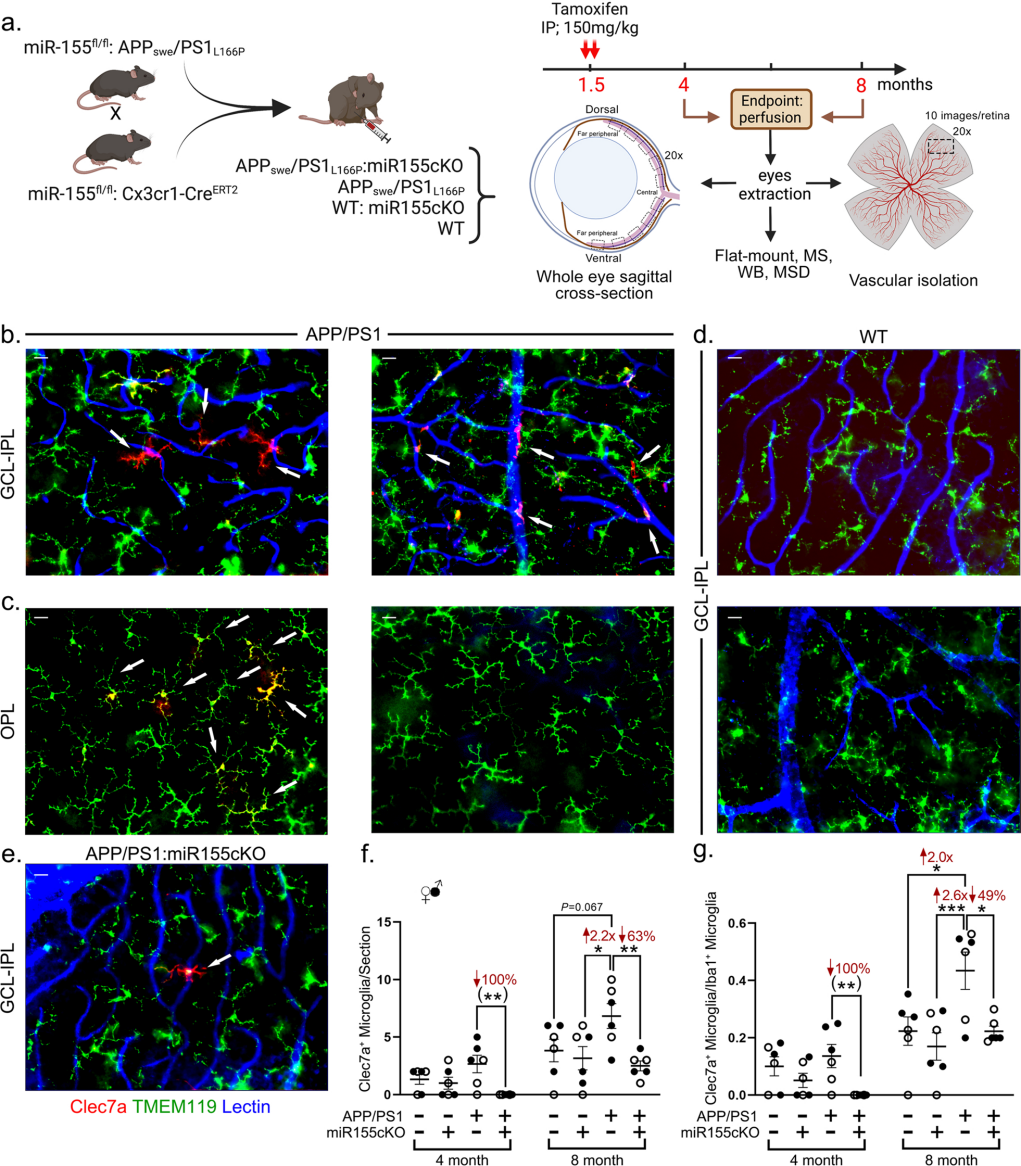
Targeting microglial miR-155 diminished the population of Clec7a^+^ MGnD microglia. **a** Schema for generation of wild type (WT) and Alzheimer’s disease (AD) mice that were specifically targeted for miR-155 in microglia. miR-155^ fl/fl^:APP/PS1 mice and miR-155^ fl/fl^:Cx3cr1^Cre^^ERT2^ mice were crossed to create APP/PS1:miR155cKO mice. Other experimental groups include APP/PS1, WT:miR155cKO and WT. Two consecutive doses of tamoxifen (150 mg/kg) were introduced peritoneally to 1.5-month-old mice to induce conditional microglial miR-155 knock-out. Mice were sacrificed at 4 months or 8 months for cross-section, vascular isolation, flat mount, mass spectrometry (MS), western blotting (WB) and Meso Scale Discovery (MSD). This illustration was created by using Biorender.com. **b**–**e** Representative images of immunofluorescent staining for Clec7a^+^ microglia (red), Tmem119^+^ microglia (green) and lectin for blood vessels (blue) on retinal flat mounts from **b** APP/PS1 genotype, ganglion cell layer (GCL) to inner plexiform layer (IPL); **c** APP/PS1 genotype, outer plexiform layer (OPL); **d** WT genotype, GCL to IPL; and **e** APP/PS1:miR155cKO genotype, GCL to IPL. Images were obtained using 20 × microscope objectives. Arrows indicate Clec7a^+^ microglia. Scale bars = 10 µm. **f** Manual counting of Clec7a^+^ microglia in each entire retinal cross-section from all experimental groups (n = 48 total, n = 6 each group). **g** Ratio of Clec7a^+^ microglia to Iba1^+^ microglia from the same mouse cohort. Data from individual mice (circles) as well as group means ± SEMs are shown. Black-filled circles represent male and clear circles represent female animals. *p < 0.05, **p < 0.01, ***p < 0.001, by three-way ANOVA with Tukey’s post-hoc multiple comparison test. Two group statistical analysis was performed using an unpaired two-tailed Student t-test and is shown in parentheses. Fold changes and percentage decreases are shown in red

All microglial counting and stereological quantification were conducted in a masked fashion. For Figs. 1f-g and Additional file 1: Fig. S5c, d, Clec7a^+^/Iba1^+^ and Iba1^+^ microglia were manually counted with the aid of the Axio Imager Z1 microscope throughout one entire retinal cross-section of each mouse from dorsal to ventral and central to peripheral retina. Each datapoint represents counting in each mouse.

**Fig. 2:**
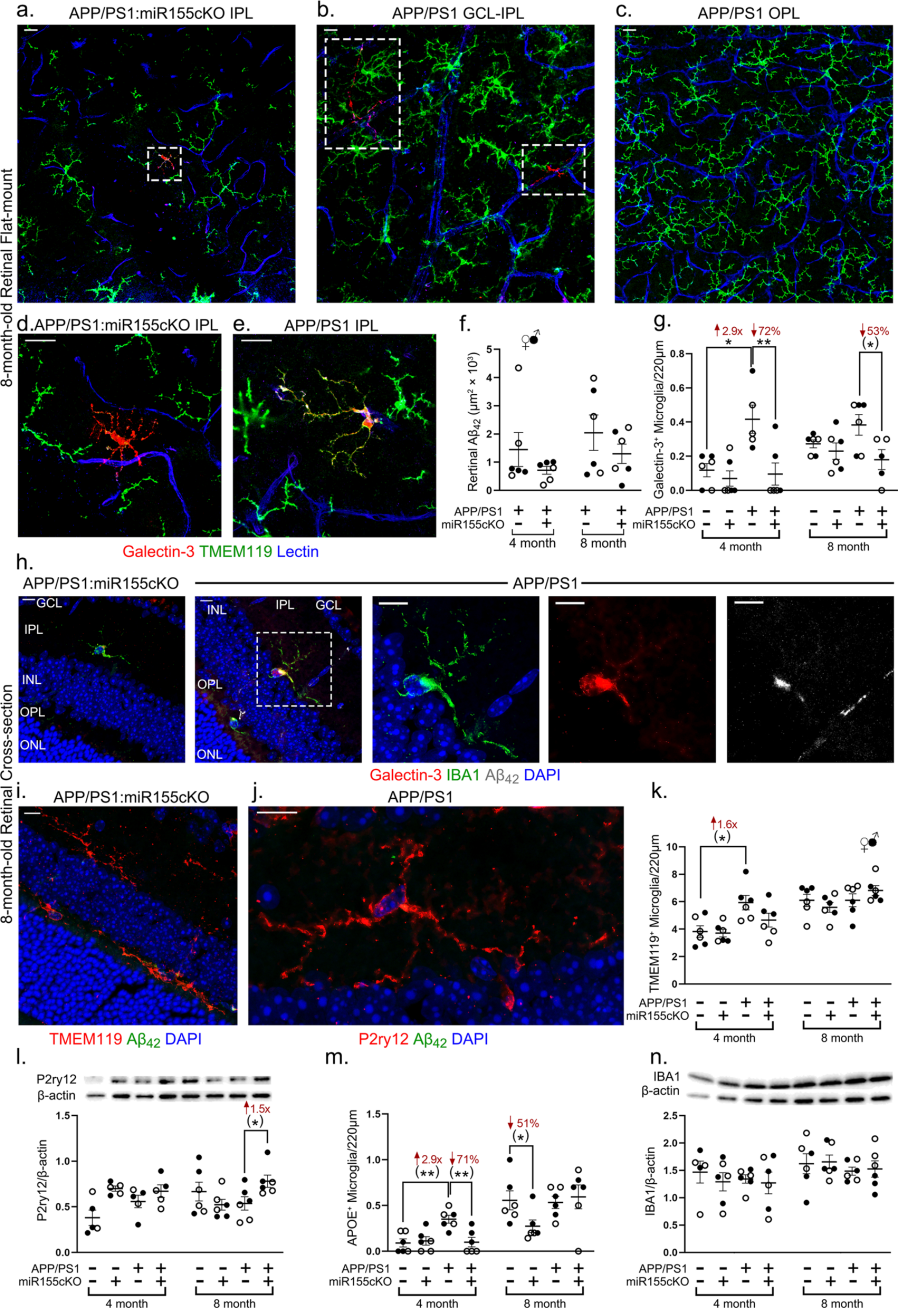
Conditional depletion of microglial miR-155 decreases Galectin-3^+^ microglia and upregulates homeostatic P2ry12 expression in APP/PS1 mice retinas. **a**–**e** Representative images of immunofluorescent staining for Galectin-3^+^ microglia (red), Tmem119^+^ microglia (green) and lectin for blood vessels (blue) on retinal flat mounts from **a** APP/PS1:miR-155cKO genotype, inner plexiform layer (IPL); **b** APP/PS1 genotype, ganglion cell layer (GCL) to IPL; **c** APP/PS1 genotype, outer plexiform layer (OPL); **d** APP/PS1:miR-155cKO genotype, IPL; and **e** APP/PS1 genotype, IPL. Images were obtained using 20 × or 63 × microscope objectives. Dashed-line rectangles highlight Galectin-3^+^ microglia. Scale bars = 10 µm. **f** Quantitative analysis of 12F4 for Aβ_42_ immunoreactivity (IR) in retinal cross-sections of mice from all experimental groups (n = 48 total, n = 6 each group). **g** Manual counting of Galectin-3^+^ microglia in each retinal cross-section from all experimental groups of the same mouse cohort. **h** Representative images of immunofluorescent staining for Galectin-3^+^ microglia (red), Iba1^+^ microglia (green), 12F4 for Aβ_42_ (white) and DAPI (blue) on retinal cross-sections from APP/PS1:miR-155cKO and APP/PS1 mice. Images were obtained using 40 × microscope objectives. Dashed-line rectangle highlights a Galectin-3^+^ microglia engulfing Aβ_42_. Scale bars = 10 µm. **i**, **j**. Representative images of eye cross-section depicting immunofluorescent staining for **i** Tmem119^+^ microglia (red), 12F4 for Aβ_42_ (green) and DAPI (blue) from an APP/PS1:miR-155cKO mouse and **j** P2ry12^+^ microglia (red), 12F4 for Aβ_42_ (green) and DAPI (blue) from an APP/PS1 mouse. **k** Manual counting of Tmem119^+^ microglia in each retinal cross-section from all experimental groups of the same mouse cohort. **l** Densitometric analysis of western blotting protein bands of P2ry12 normalized by β-actin control for retinal lysates from all experimental groups (n = 48 total, n = 6 each group). **m** Manual counting of Apoe^+^ microglia in each retinal cross-section from all experimental groups of the same mouse cohort shown in figures **g** and **k** **n** Densitometric analysis of western blotting protein bands of Iba1 normalized by β-actin control for retinal lysates from all experimental groups of the same cohort as figure **l.** Data from individual mice (circles) as well as group means ± SEMs are shown. Black-filled circles represent male and clear circles represent female animals. *p < 0.05, **p < 0.01, by two-way or three-way ANOVA with Tukey’s post-hoc multiple comparison test. Two group statistical analysis was performed using an unpaired two-tailed Student t-test and is shown in parentheses. Fold changes and percentage decreases are shown in red

For stereological quantifications of Aβ_42_ in Fig. 2f, claudin-1 in Fig. 5b, ZO-1 in Fig. 5e, Aβ_40_ in Fig. 5f and total Aβ in Fig. 5h, the fluorescence of specific signals was captured using the same setting and exposure time for each image and mouse with the aid of the Axio Imager Z1 microscope (with motorized Z-drive) with an AxioCam MRm monochrome camera (version 3.0; at a resolution of 1388 × 1040 pixels, 6.45 µm × 6.45 µm pixel size, and a dynamic range of > 1:2200, which delivers low-noise images due to a Peltier-cooled sensor). For Fig. 5, ten 20 × Z-stack images at 15 µm thickness were obtained randomly from each entire isolated retinal vascular network. For quantification of Aβ_42_ in Fig. 2f, ten 20 × images were obtained throughout dorsal to ventral and central to peripheral retina from one cross-section of each mouse (Fig. 1a, dashed-line rectangles). Acquired images were converted to gray scale and standardized to baseline using a histogram-based threshold in the NIH ImageJ software program (version 1.52o). For each biomarker, the total area of immunoreactivity was determined using the same threshold percentage from the baseline in ImageJ (with the same percentage threshold setting for all diagnostic groups). The images were then subjected to particle analysis to determine the immunoreactive (IR) area. For all IR analyses in ImageJ, under ‘Analyze—Set Scale’ function, we set the ‘Distance in pixels’ to 150, ‘Known distance’ to 0, ‘Pixel aspect ratio’ as 1, ‘Unit of length’ as inch. Then we used the “Analyze particles” function with default setting to analyze our images. Each datapoint was calculated by averaging the values per mouse.

For Fig. 2g, k, m and Additional file 1: Fig. S5a, b and e–h, ten images were obtained throughout dorsal to ventral and central to peripheral retina (Fig. 1a, dashed-line rectangles) from one cross-section of each mouse for each biomarker by using the Axio Imager Z1 microscope described above with the 20 × objective. Then, Galectin-3^+^, Apoe^+^ and Tmem119^+^ microglia were manually counted in each image (approximate retinal length ~ 220 µm). Each datapoint represents the average value of microglial counting from 10 images of each mouse retina.

### Statistical analysis

GraphPad Prism version 8.3.0 (GraphPad Software) was used for the analyses. Groups with two or three independent variables/factors were analyzed by using two-way ANOVA or three-way ANOVA followed by Tukey’s multiple comparison test to understand the interaction between the two or three independent variables. Two-group comparisons were analyzed using a two-tailed unpaired Student t-test. The statistical association between two or more variables was determined using Pearson’s correlation coefficient (*r*) test (Gaussian-distributed variables; GraphPad Prism). Pearson’s *r* indicates the direction and strength of the linear relationship between two variables. Required sample sizes for two group (differential mean) comparisons were calculated using the nQUERY t-test model, assuming a two-sided α level of 0.05, 80% power, and unequal variances, with the means and common standard deviations for the different parameters. Results are expressed as means ± SEMs. A *P* value less than 0.05 is considered significant.

## Results

### Conditional deletion of microglial miR-155 reduces retinal Clec7a^+^ MGnD in APP/PS1 mice

To investigate the impact of microglial miR-155 in the retinas of WT and AD-model mice, we generated multiple mouse lines (Fig. 1a). All resulting mice were sacrificed at an early-disease stage of 4 months or an early-symptomatic-disease age of 8 months. The animals’ eyes were extracted for cross-section, retinal vascular isolation, retinal flat mounts, mass spectrometry proteomics analysis, and analyses by western blot or MSD of inflammatory profiles. Due to the limited mouse retina tissue size, assessment of miR-155 expression in microglia was conducted on isolated microglial cells from mouse brains, and analyzed by qRT-PCR to validate the conditional miR-155 knock-out model. In both 4-month-old and 8-month-old mouse groups, our analyses demonstrated substantially decreased microglial miR-155 expression in APP/PS1 mice with conditional miR-155 knock-out compared to APP/PS1 mice (Additional file 1: Fig. S1).

Recently, Krasemann et al. demonstrated a unique population of Clec7a^+^/P2ry12^–^ MGnD microglia that is closely associated with neuritic Aβ plaques in the AD mouse cortex [47]. Since miR-155 was identified as a post-translational regulator of MGnD phenotype expression, we first detected protein Clec7a expression in retinal flat mounts to evaluate the microglial phenotype in WT and APP/PS1 mice with or without microglial miR-155 expression. Clec7a staining was combined with transmembrane Protein 119 (Tmem119), a microglia-specific transmembrane protein that is highly enriched in homeostatic microglia [10, 43, 47]. In the retinal ganglion cell layer (GCL) and the inner plexiform layer (IPL) of 8-month-old APP/PS1 mice, we observed an abundance of Clec7a^+^/Tmem119^–^ microglia that are touching or adhering to retinal blood vessels, as seen by lectin staining (Fig. 1b). In contrast, a population of double positive Clec7a^+^/Tmem119^+^ microglia was seen in the outer plexiform layer (OPL) and was not attached to retinal blood vessels (Fig. 1c). No Clec7a^+^ microglia were detected in 8-month-old WT mice (Fig. 1d). Importantly, only a single Clec7a^+^/TMEM119^–^ microglial cell was detected in the representative APP/PS1:miR-155cKO mouse (Fig. 1e).

To validate our observations in retinal flat mounts, we next analyzed eye cross-sections (n = 48 total, n = 6 mice per experimental group) immunostained for Clec7a and Iba1 – the ionized calcium-binding adaptor protein-1, which is a constitutive and specific marker of all resting and activated myeloid cells. We quantified the number of Clec7a^+^ microglia in all genotype groups (see representative images in Additional file 1: Fig. S2a–c). A manual count of microglia revealed trends of increased Clec7a^+^/Iba1^+^ microglial cells in the retinas of APP/PS1 mice compared to those of WT control mice at 4 and 8 months of age (Fig. 1f). Conditional knock-out of miR-155 in microglia significantly reduced Clec7a^+^/Iba1^+^ microglia to levels equal to or below those detected in the WT mice. A comparison of ratios of Clec7a^+^ microglia to Iba1^+^ total microglia population indicated a significant increase in retinal Clec7a^+^ microglia in 8-month-old APP/PS1 mice versus age-matched WT mice and substantial downregulation of Clec7a^+^ microglia in response to miR-155cKO in both 4- and 8-month-old APP/PS1 mice (Fig. 1g).

### Conditional deletion of microglial miR-155 downregulates retinal microglial Galectin-3^+^ while restoring P2ry12^+^ expression

Galectin-3 is another marker that is strongly expressed in plaque-associated MGnD microglia [47]. Recent studies in mouse models have demonstrated that Galectin-3 is involved in multiple inflammatory processes in diverse neurological diseases [83, 86] and that it promotes Aβ toxicity and oligomerization in AD [8, 87]. We immunostained against Galectin-3 (red) and Tmem119 (green) to evaluate the effects of miR-155cKO on the Galectin-3^+^ microglial population. In retinal flat mounts from both APP/PS1 and APP/PS1:miR-155cKO mice, we detected Galectin-3^+^ microglia in different cell layers (Fig. 2a–e; extended representative and quantitative data are included in Additional file 1: Figs. S3–S5). We detected a single Galectin-3^+^ microglia in the IPL of a representative APP/PS1:miR-155cKO mouse (Fig. 2a), while multiple Galectin-3^+^ microglia were observed extending from the GCL to the IPL in a representative APP/PS1 mouse (Fig. 2b). Like Clec7a^+^/Tmem119^–^ microglia, Galectin-3^+^/Tmem119^–^ microglia were adhering to the retinal blood vessels, and microglial cell processes were found to surround or extend along the blood vessels (Fig. 2d, Additional file 1: Fig. S3a). Unlike Clec7a^+^ microglial staining, Galectin 3^+^ microglia were not detected in the retinal OPL (Fig. 2c, Additional file 1: Fig. S3a, right image**)**. Instead, abundant numbers of Tmem119^+^ homeostatic microglia were present in the OPL. Interestingly, we also observed a double-positive Galectin-3^+^ Tmem119^+^ in the IPL of an APP/PS1 mouse (Fig. 2e). Our qualitative observations in retinal flat mounts regarding layer distribution of MGnD microglia were further supported by quantitative microglial counting analyses in retinal cross-sections (Additional file 1: Fig. S5). Stereological quantification of the 12F4-positive Aβ_42_ burden in the retina of APP/PS1 mice suggested that there is no significant change in total Aβ_42_ deposition with miR-155cKO when the animals are 8 months old (Fig. 2f).

To further quantify Galectin-3^+^ microglia in the retina and evaluate the effects of miR-155cKO in microglia, we performed immunostaining for Galectin-3 (red) and Iba1 (green) on eye cross-sections (n = 48 total, n = 6 each experimental group) (Fig. 2g and h). Manual counting demonstrated a significantly reduced amount of Galectin-3^+^ microglia in APP/PS1:miR-155cKO mice compared to that in APP/PS1 mice, similar to changes observed in Clec7a^+^ microglia (Fig. 2g). The Galectin-3^+^ microglia engulfed retinal Aβ_42_ plaques in a representative APP/PS1 mouse (Fig. 2h). Assessment of whole retinal Galectin-3 expression levels by western blot revealed no significant change due to miR-155cKO in microglia (Additional file 1: Fig. S3b).

Tmem119 and P2ry12 were both previously identified as markers for homeostatic microglia [47]. Loss of P2ry12 and Tmem119 were found to negatively affect microglial survival and phagocytic activity [10]. To evaluate whether miR-155cKO in microglia changed the homeostatic microglial population in the retina, we further immunostained for Tmem119 and P2ry12 in eye cross-sections in the same mouse cohort (Fig. 2i, j; see extended images in Additional file 1: Fig. S4a and b). Manual counting of Tmem119^+^ microglia demonstrated an early significant increase in the APP/PS1 mice, indicating upregulated inflammatory activity in the retinas of 4-month-old APP/PS1 mice compared to that in the retinas of WT controls (Fig. 2k). Although miR-155cKO did not appear to have effects on Tmem119^+^ microglia, western blot analysis detected an overall significant 1.5-fold increase in the expression of P2ry12 in 8-month-old APP/PS1:miR-155cKO mice compared to APP/PS1 mice (Fig. 2l). Apoe is another major upstream regulator of MGnD microglia, which is tightly associated with an increased risk for AD and is upregulated in the MGnD/DAM phenotype [43, 47]. Examination of Apoe expression in retinal microglia by immunostaining of eye cross-sections in this mouse cohort (Additional file 1: Fig. S4c) suggests an early and significant increase in the number of Apoe^+^ microglia in 4-month-old APP/PS1 mice versus WT controls, which is downregulated by miR-155cKO (Fig. 2m). Intriguingly, manual counting of Apoe^+^ microglia in the 8-month-old age group revealed significant decreases due to miR-155cKO only in the WT mice (Fig. 2m). Overall, Iba1 expression by western blot shows no significant differences in any experimental group (Fig. 2n), suggesting no changes in total retinal myeloid cell population. Microglial quantitation per marker in separate retinal layers indicated that the greatest microglial phenotypic shift occurs within the GCL and IPL but not in the OPL (Additional file 1: Fig. S5a–h).

### Conditional deletion of microglial miR-155 decreases pro-inflammatory cytokines in APP/PS1 mice

**Fig. 3:**
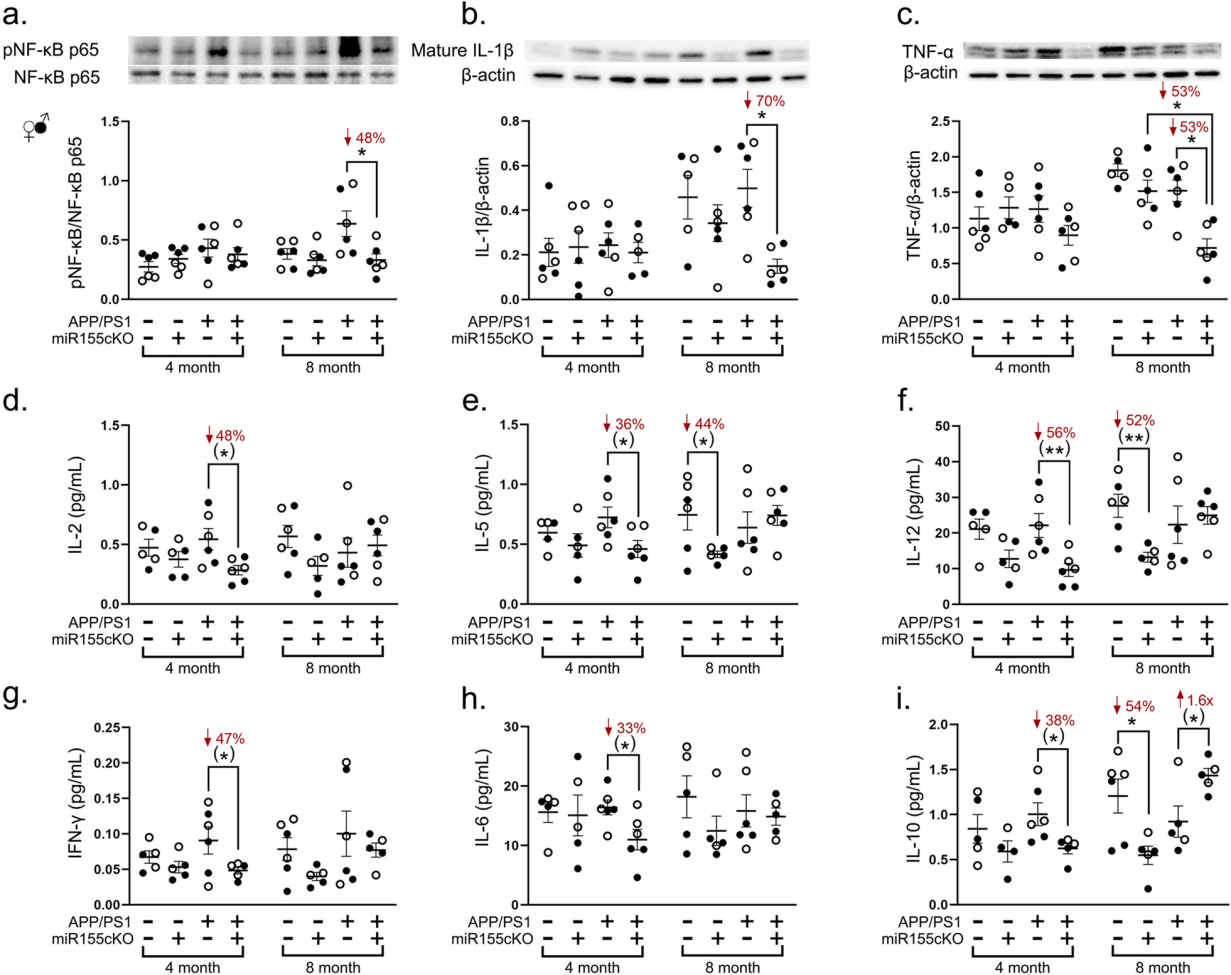
Targeting microglial miR-155 ameliorates inflammation in APP/PS1 mouse retinas. **a**–**c** Densitometric analyses of western blotting protein bands of **a** phosphorylated NF-κB p65, **b** mature IL-1β, and **c.** TNF-α normalized by **a** total NF-κB p65 or **b** and **c** β-actin control for retinal lysates from all experimental groups (n = 48 total, n = 6 each group). **d**–**i** Meso Scale Discovery analysis for protein expression of cytokines including **d** IL-2, **e** IL-5, **f** IL-12, **g** IFN-γ, **h** IL-6 and **i** IL-10 from the same mouse cohort. Data from individual mice (circles) as well as group means ± SEMs are shown. Black-filled circles represent male and clear circles represent female animals. *p < 0.05, **p < 0.01, by three-way ANOVA with Tukey’s post-hoc multiple comparison test. Two group statistical analysis was performed using an unpaired two-tailed Student t-test and is shown in parentheses. Fold changes and percentage decreases are shown in red

The decreased population of neurodegeneration-related MGnD and the upregulated homeostatic microglial marker P2ry12 in APP/PS1 mice in response to targeted miR-155cKO in microglia prompted further investigation into the extent of retinal inflammation in these mice. Whole retinal lysates were processed for western blotting of regulatory inflammatory markers and MSD of multiple cytokines (n = 48 mice total; n = 6 mice per experimental group). Nuclear factor kappa B (NF-κB) p65 subunit, interleukin (IL)-1β and tumor necrosis factor (TNF)-α are all pivotal upstream pro-inflammatory mediators. Densitometric analysis of western blot demonstrated a significant reduction in phosphorylation of the retinal NF-κB p65 subunit, as well as decreased levels of mature IL-1β and TNF-α following miR-155cKO in microglia in the 8-month-old APP/PS1 mice (Fig. 3a-c). High-sensitivity measurement of cytokine concentrations, obtained by MSD, revealed early and consistent decreases in IL-2, IL-5, IL-12, interferon-gamma (IFN-γ), IL-6 and IL-10 expression in APP/PS1:miR-155cKO versus APP/PS1 mice 4 months of age (Fig. 3d–i). Remarkably, miR-155cKO in microglia restored expression of the immunoregulatory cytokine IL-10 in the retinas of APP/PS1 mice when 8 months of age (Fig. 3i).

### Targeting microglial miR-155 enhances PI3K-Akt signaling pathways and decreases Spp1 expression in retinas of APP/PS1 mice

**Fig. 4:**
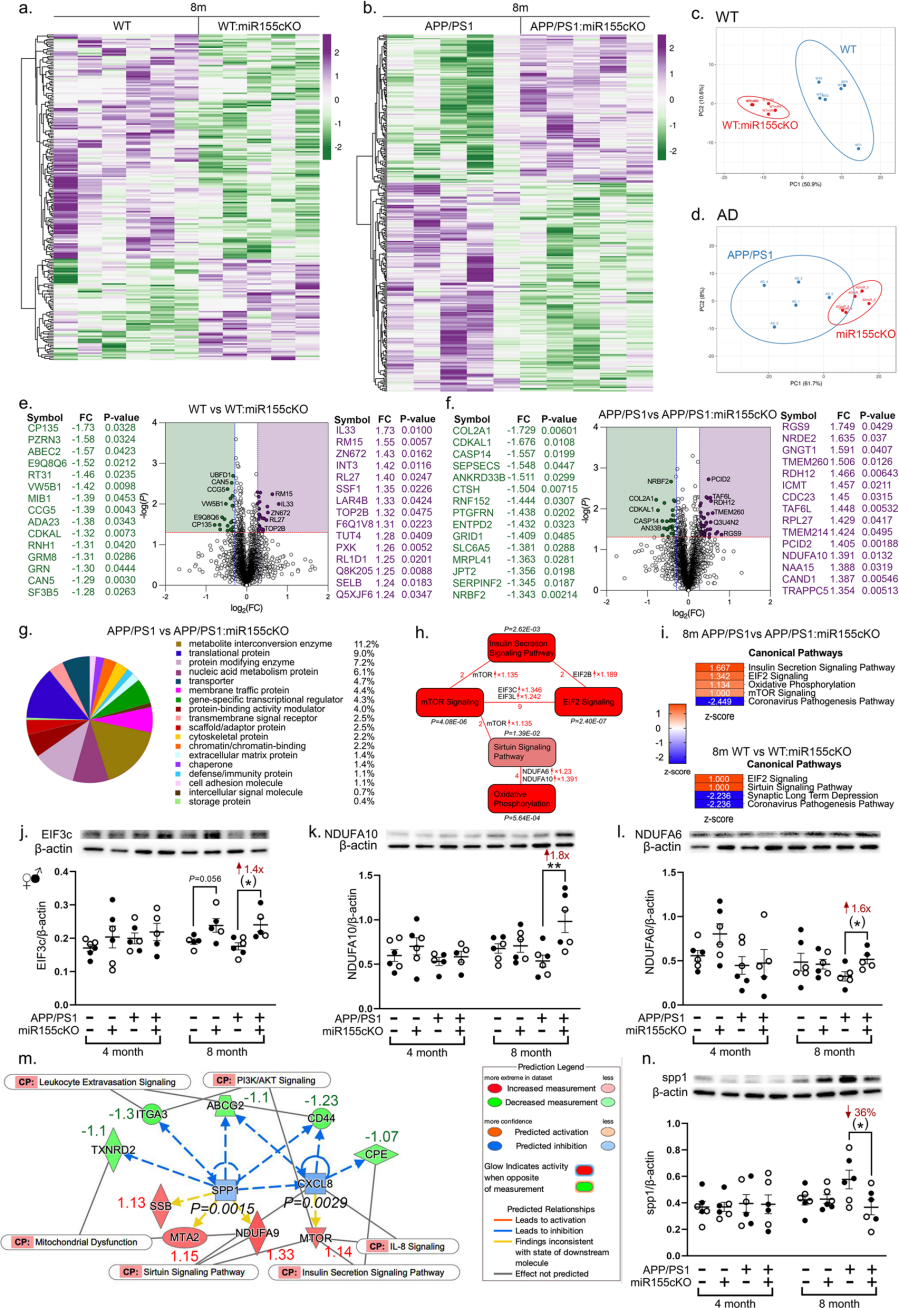
Conditional microglial miR-155 knock-out enhanced PI3K-Akt signaling in APP/PS1 mouse retinas. **a** Detectable protein hierarchies displayed as heatmaps from **a** a comparison of 8-month-old WT and WT:miR-155cKO mice and **b** a comparison of 8-month-old APP/PS1 and APP/PS1:miR-155cKO mice; upregulated proteins are shown in purple and downregulated proteins in green. **c**, **d** Principal component analysis for **a** and **b e**–**f** Volcano plots and top 15 up- or downregulated proteins by fold change between **e** 8-month-old WT vs. WT:miR-155cKO mice and **f** 8-month-old APP/PS1 vs. APP/PS1:miR-155cKO mice; upregulated proteins are shown in purple and downregulated proteins in green. **g** Pie chart of PANTHER functional classification analysis showing fraction and percentage of significantly differentially expressed proteins (DEPs, up- or downregulated proteins) grouped by protein class category based on a comparison of APP/PS1 and APP/PS1:miR-155cKO mice. **h** Ingenuity pathway analysis (IPA) of canonical pathways based on APP/PS1 vs. APP/PS1:miR-155cKO mice. Several upregulated PI3K-Akt pathways are shown here. *P* values are labelled on each pathway. Quantities of commonly changed molecules between each two pathways are labelled in red together with highlighted molecules and detectable fold changes. **i** Z-scores for the IPA analysis of canonical pathways from 8-month-old WT vs. WT:miR-155cKO mice and 8-month-old APP/PS1 and APP/PS1:miR-155cKO mice. **j**–**l** Densitometric analysis of western blotting protein bands of **j** EIF3c, **k** NDUFA10, and **l** NDUFA6, each normalized by β-actin control for retinal lysates from all experimental groups (n = 48 total, n = 6 each group). **m** IPA analysis for upstream regulators Spp1 and Cxcl-8 based on 8-month-old APP/PS1 vs. APP/PS1:miR-155cKO versus mice. Refer to “prediction legend” in the graph for details. Detectable fold changes of DEPs are written next to each molecule. CP-common pathways. **n** Densitometric analysis of western blotting protein bands of SPP1 normalized by β-actin control for retinal lysates from the same cohort. Data from individual mice (circles) as well as group means ± SEMs are shown. Black-filled circles represent male and clear circles represent female animals. *p < 0.05, **p < 0.01, by three-way ANOVA with Tukey’s post-hoc multiple comparison test. Two group statistical analysis was performed using an unpaired two-tailed Student t-test and is shown in parentheses. Fold changes and percentage decreases are shown in red

To identify the molecular mechanisms by which miR-155cKO in microglia affects the WT and AD-model retina, we initiated a global protein expression analysis utilizing mass spectrometry (MS). The MS analysis was conducted on retinal lysates from the aforementioned mouse cohort used for inflammatory markers (n = 48 mice total; n = 6 mice per experimental group). Hierarchical heatmaps of all significantly altered proteins are presented to demonstrate the global effects of miR-155cKO in microglia in 8-month-old WT:miR-155cKO mice compared to WT mice as well as in APP/PS1:miR-155cKO mice compared to APP/PS1 mice (Fig. 4a and 4b), respectively; extended data for 4- and 8-month-old mice are shown in Additional file 1: Fig. S6a and b and Additional file 1: Tables S1–S8. Principal component analyses further verified separate protein signature profiles for mice with and without miR-155cKO targeted to microglia in the retina (Fig. 4c and d). Volcano plots provide a higher resolution analysis of the top up- and down-regulated differentially expressed proteins (DEPs), based on the fold-change and the most significant *P* values (Fig. 4e and f). Proteomics analysis for the effect of miR-155cKO in the retinas of 4-month-old mice showed a clear separation of protein expression in both WT groups as well as in the APP/PS1 group (Additional file 1: Fig. S6c, d). Most notable DEPs in 4-month-old APP/PS1:miR-155cKO mice versus APP/PS1 controls were m6A methylation related molecules. We found commonly upregulated protein levels of mettl3, mettl14, and mettl16 (Additional file 1: Figs. S6g–i). PANTHER functional analysis categorized DEPs based on protein functions and demonstrated that the largest changes were in proteins related to the metabolite interconversion enzyme and in translational proteins (Fig. 4g, Additional file 1: Fig. S7a, see PANTHER function analysis for 4-month-old mice in Additional file 1: Fig. S6e, f).

Next, we conducted an IPA to determine significantly changed canonical pathways due to microglia-targeted miR-155cKO in the retina. This analysis revealed enhanced signaling of multiple PI3K-Akt related pathways, including the insulin secretion, mTOR, EIF2, and sirtuin signaling pathways, in conditionally miR-155 depleted microglia in the retinas of APP/PS1 mice (Fig. 4h). These findings suggest an overall augmented PI3K-Akt signaling cascade. Notably, oxidative phosphorylation (OXPHOS) signaling was also improved by miR-155cKO (Fig. 4i; Z-scores of canonical pathways are presented in the heatmaps). To verify the MS data and these activated pathways, we selected the most commonly upregulated DEPs, identified in Fig. 4h, and performed protein expression verification by western blot analyses. Indeed, densitometric western blot analysis validated increased expression of EIF3c (Fig. 4j, a common molecule of mTOR and EIF2 signaling pathways) as well as NDUFA10 and NDUFA6 (Fig. 4k–l; common molecules of the sirtuin and oxidative phosphorylation signaling pathways) in the retinas of 8-month-old APP/PS1 mice with microglial miR-155cKO.

Further analysis of upstream regulators, performed using IPA algorithm prediction, revealed significant downregulation of retinal Spp1 and Cxcl8 in 8-month-old APP/PS1 mice with microglial miR-155cKO (Fig. 4m). This prediction was based on a set of proteins that were related to inflammation, PI3K-Akt signaling and mitochondrial function (Fig. 4m). We then successfully validated the predicted decrease in Spp1 expression by using western blot analysis (Fig. 4n) as well as immunofluorescence staining of retinal cross-sections (Additional file 1: Fig. S7b) and flat mounts (Additional file 1: Fig. S7c and d). Intriguingly, Spp1 was predominantly expressed by retinal ganglion cells in the retinal ganglion cell layer.

### Targeting microglial miR-155 rescues tight-junction molecular integrity at the blood-retinal barrier and diminishes retinal vascular amyloidosis

**Fig. 5:**
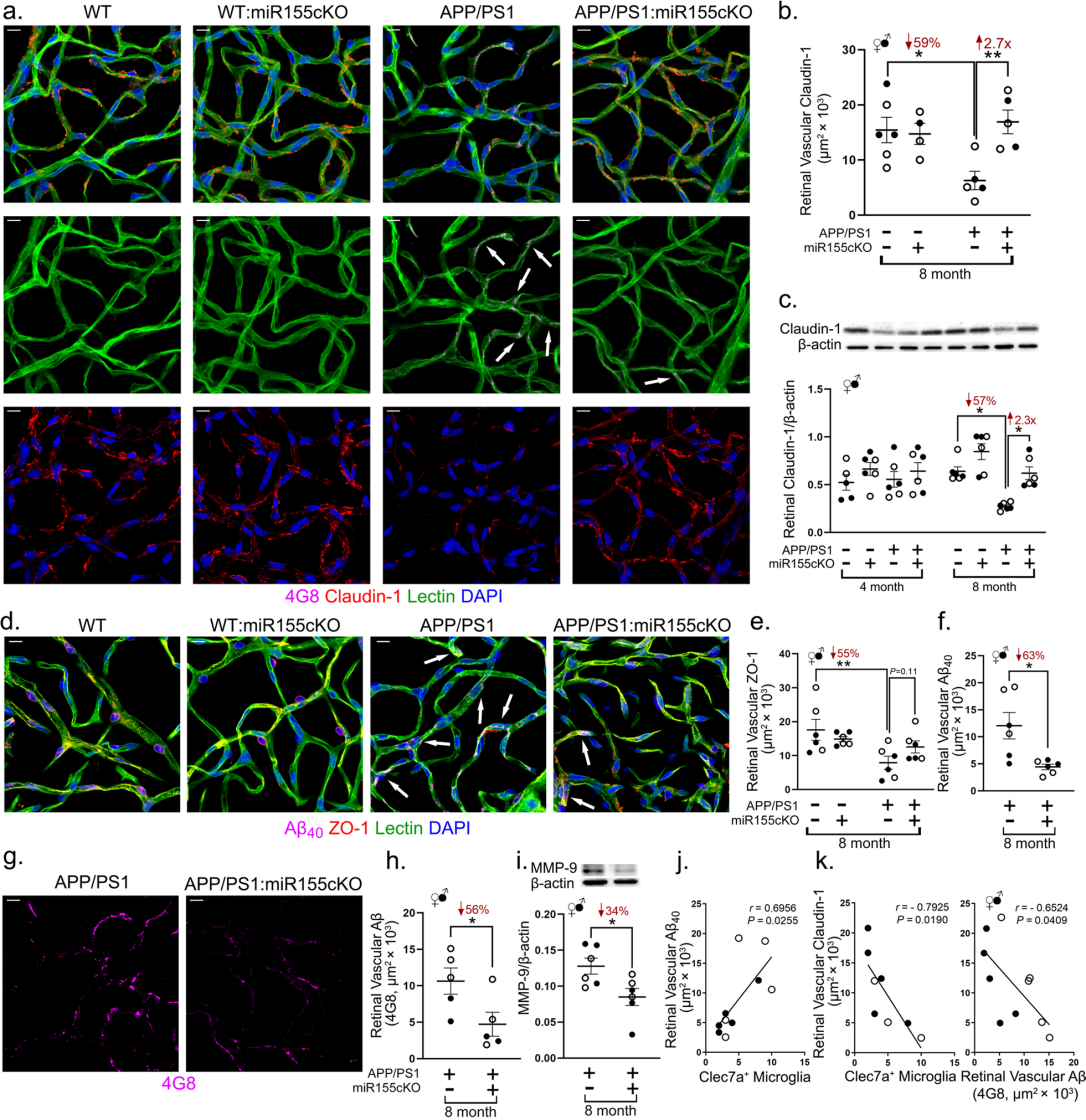
Targeting microglial miR-155 protected retinal blood vessels and reduced vascular amyloidosis in APP/PS1 mouse retinas. **a** Representative images of immunofluorescent staining for 4G8 of Aβ (magenta), Claudin-1 (red), lectin for blood vessels (green) and DAPI (blue) on isolated retinal blood vessels from all four genotypes—WT, WT:miR-155cKO, APP/PS1, and APP/PS1:miR-155cKO—in 8-month-old mice. Images were obtained using the 63 × microscope objective. Arrows indicate vascular Aβ. Scale bars = 10 µm. **b** Quantitative analysis of Claudin-1-immunoreactivity (IR) in isolated retinal blood vessels from all 8-month-old experimental groups (n = 24 total, n = 6 each group). **c** Densitometric analysis of western blotting protein bands of Claudin-1 normalized by β-actin control for retinal lysates from all experimental groups (n = 48 total, n = 6 each group). **d** Representative images of immunofluorescent staining for 11A50-B10 of Aβ_40_ (magenta), ZO-1 (red), lectin for blood vessels (green) and DAPI (blue) on isolated retinal blood vessels from all four genotypes—WT, WT:miR-155cKO, APP/PS1, and APP/PS1:miR-155cKO—in 8-month-old mice. Images were obtained using the 63 × microscope objective. Arrows indicate vascular Aβ. Scale bars = 10 µm. **e** Quantitative analysis of ZO-1-IR in isolated retinal blood vessels from same mouse cohort as shown in panel **b**. **f** Quantitative analysis of 11A50-B10 for Aβ_40_-IR in isolated retinal blood vessels from 8-month-old APP/PS1 experimental groups (n = 12 total, n = 6 each group). **g** Representative images of immunofluorescent staining of 4G8 for Aβ (magenta) on isolated retinal blood vessels from 8-month-old APP/PS1 or APP/PS1:miR-155cKO mice groups. **h** Quantitative analysis of 4G8 for Aβ in isolated retinal blood vessels from the same mouse cohort shown in panel **f**. **i** Densitometric analysis of western blotting protein bands of MMP-9 normalized by β-actin control for retinal lysates from APP/PS1:miR-155cKO vs. APP/PS1 mice (n = 12 total, n = 6 each group). **j**, **k** Pearson’s coefficient (*r*) correlation between **j.** retinal vascular Aβ_40_ and retinal Clec7a^+^ microglia, **k** retinal vascular Claudin-1-IR and retinal Clec7a^+^ microglia or retinal vascular Aβ-IR. Data from individual mice (circles) as well as group means ± SEMs are shown. Black-filled circles represent male and clear circles represent female animals. *p < 0.05, **p < 0.01, by two-way or three-way ANOVA with Tukey’s post-hoc multiple comparison test. Two group statistical analysis was performed using an unpaired two-tailed Student* t*-test. Fold changes and percentage decreases are shown in red

This study found that a persist depletion of miR-155 in microglia induced a phenotypic switch of retinal microglia that had been near or touching blood vessels. Further, a previous study reported a protective effect of miR-155 on endothelial tight junctions in an in vitro ischemic stroke cell model [67]. Our earlier studies in another double transgenic APP/PS1 mouse model (APP_SWE_/PS1_ΔE9_) revealed early and progressive downregulation of Claudin-1 and ZO-1, both of which are key inner blood-retinal barrier (iBRB) tight junction molecules [78]. Hence, we next investigated whether miR-155cKO could protect TJs in the BRB of the current APP/PS1 mouse model. We isolated retinal vascular networks from 8-month-old mice (n = 24 mice total; n = 6 mice per experimental group) and performed immunofluorescence staining against Claudin-1 (red), Aβ (clone 4G8; magenta), retinal blood vessels (lectin; green) and nuclei (DAPI; blue), as seen in Fig. 5a. We discovered a substantial reduction in retinal vascular Claudin-1 along with an intense deposition of vascular Aβ in APP/PS1 mice versus WT controls, which was reversed by miR-155cKO (Fig. 5a). Both stereological quantification of retinal vascular Claudin-1 (Fig. 5b) and western blot analysis of total retinal Claudin-1 levels (Fig. 5c) indicated significantly improved Claudin-1 expression following miR-155cKO in APP/PS1 mice, which validated our observation. We also performed immunostaining of retinal vascular ZO-1 (red), Aβ_40_ (clone 11A50-B10; magenta), lectin (green) and DAPI (blue) in the same cohort (Fig. 5d, Additional file 1: Fig. S8a). Stereological quantification uncovered a prominent downregulation of retinal vascular ZO-1 in 8-month-old APP/PS1 mice versus WT controls, while miR-155cKO displayed a nonsignificant trend of restoring retinal vascular ZO-1 expression (Fig. 5e).

Further immunostaining against Aβ deposits in retinal vascular networks followed by stereological quantification indicated significant decreases in both 4G8^+^ total Aβ burden and 11A50-B10^+^ Aβ_40_ burden (Fig. 5f–h, Additional file 1: Fig. S8b) in 8-month-old APP/PS1 mice bearing the miR-155cKO in microglia. Interestingly, western blot analysis of retinal matrix metallopeptidase 9 (MMP-9), a TJ-degrading enzyme that also regulates Spp1 processing [53, 95], found to be increased in 8-month-old APP/PS1 mice versus WT controls. Elevated MMP-9 expression was abrogated by microglial miR-155cKO (Fig. 5i, Additional file 1: Fig. S9a). Finally, Pearson’s (*r*) correlation analysis revealed that retinal vascular Aβ_40_ and Claudin-1 expression are tightly associated with quantities of Clec7a^+^ microglia, but not with Galectin-3^+^ microglia in 8-month-old APP/PS1 mice (Fig. 5j and k, Additional file 1: Fig. S9b-g). We also observed an inverse correlation between vascular 4G8^+^ Aβ burden and Claudin-1 expression in isolated retinal blood vessels of APP/PS1 mice (Fig. 5k, right graph). In contrast, retinal vascular ZO-1 did not correlate with retinal vascular Aβ_40_ burden (Additional file 1: Fig. S9h).

## Discussion

In this study we identified Clec7a^+^ and Galectin-3^+^ MGnD microglial populations in AD-model retinas, which were increased during disease progression and often associated with retinal blood vessels. These findings suggest an enhanced phenotypic switch of retinal microglia toward the MGnD phenotype that is associated with AD pathogenesis. Essentially, we have provided original evidence that the neurodegeneration- and inflammation-promoting MGnD microglial phenotype plays a role in mediating microvasculopathy. We also found that knockout of microglial miR-155 diminished both Clec7a^+^ and Galectin-3^+^ MGnD populations while promoting expression of the homeostatic microglial marker, P2ry12. Results from this study propose a new direction for reducing retinal inflammation and vascular damage via inhibition of MGnD microglial expression by targeting miR-155.

Clec7a is a non-toll like receptor pattern recognition receptor that is expressed on multiple types of immune cells including microglia [9]; there is evidence that it supports regulation of phagocytic activity in retinal microglia [56]. In AD mouse brains, Clec7a was found to be significantly upregulated in MGnD microglia and associated with neuritic plaques and neurodegeneration [47]. Here, we observed increased Clec7a^+^ microglia in retinas obtained from APP/PS1 mice, in comparison to retinas from WT mice, in agreement with the previous brain study in AD mouse models [47]. Dysregulated retinal microglia secrete many inflammatory mediators that can damage TJs and BRB structural and functional integrity [16, 72, 73]. Since Cx3cr1 CreERT2 targeting does not distinguish between microglia and perivascular macrophages, it is also possible to attribute these effects to changes in the phenotype of these macrophages. In this study, we found that most Clec7a^+^ microglia reside close to and along retinal microvessels across the GCL and IPL in APP/PS1 mice. Accordingly, Pearson’s correlation analysis showed a strong inverse association in APP/PS1 mice between the frequency of retinal Clec7a^+^ microglia and expression of vascular Claudin-1, an important TJ component of the BRB. These results may therefore suggest a role for Clec7a^+^ microglia in inducing damage to TJs and the BRB in AD-model mice.

As it relates to Galectin-3, a study of ischemic brain injury described its essential role in acute-phase cerebral microglia activation [49]. In the brains of AD-model mice, recent scRNAseq studies revealed that Galectin-3 is highly expressed in chronically activated MGnD microglia [37, 47]. Boza-Serrano et al. further confirmed that Galectin-3^+^ microglia are typically found surrounding Aβ plaques in brain cortices from both AD patients and 5xFAD mice [8]. Here, our analyses identified a population of Galectin-3^+^ microglia in AD mice retinas. Of note, immunohistochemistry analyses in previous reports showed extracellular expression of Galectin-3 in mouse retinas [52, 57]. Our western blot analyses did not detect a difference of whole retinal Galectin-3 levels among the groups, suggesting that the change of Galectin-3 expression in microglia-specific immunohistochemistry was primarily in MGnD microglia based on manual counting analysis. Interestingly, we mainly observed Galectin-3^+^ microglia in inner retinal layers, suggesting that, unlike Clec7a^+^ microglia, Galectin-3^+^ microglia do not migrate to outer retinal layers. Although retinal Galectin-3^+^ microglia were located close to blood vessels on flat mounts, our correlation analyses did not identify relationship between the abundance of Galectin-3^+^ microglia and loss of retinal vascular TJ expression. Intriguingly, a previous study found that genetic knock-out of Galectin-3 improved microvascular leakage and TJ integrity in streptozotocin-induced type I diabetic mice [13]. Future studies should apply quantitative methods to further explore the location and impact of MGnD vs. homeostatic microglia on retinal blood vessels in different disease models.

Although both Clec7a^+^ and Galectin-3^+^ microglia were previously found to accumulate around Aβ plaques in the AD mouse brain [8, 47], how they regulate Aβ formation and clearance has yet to be determined. A recent report described an intriguing effect of Galectin-3–promoting Aβ aggregation via the TREM2 pathway in the mouse hippocampus [8]. Here, miR-155cKO significantly ameliorated retinal vascular Aβ_40_ load as well as general vascular Aβ, whereas it did not modify abluminal Aβ_42_ levels in retinas from APP/PS1 mice. We further observed that MGnD microglia are increased in retinas of AD-model mice. They often resided close to and in direct contact with blood vessels, and Clec7a^+^ MGnD microglia exhibited a direct correlation with vascular Aβ_40_ burden. Hence, we concluded that increased MGnD microglia are associated with retinal vascular pathology in AD-model mice. Future studies should quantitatively explore the relationship between MGnD vs. homeostatic microglia and retinal vascular pathology in AD patients. Of note, another recent study revealed a neuroprotective role for the MGnD/DAM marker TREM2 in mediating microglial phagocytosis of TDP-43 during neurodegeneration [94], indicating that MGnD/DAM microglia are also essential for maintaining immune system homeostasis and thereby blood-barrier integrity.

It is widely perceived that tissue damage due to reactive microglial activation is primarily mediated through involvement in chronic inflammation [5, 85] by secretion of a host of pro-inflammatory cytokines and chemokines that are toxic to neurons [84]. In the current study, early versus later changes in molecular inflammatory mediators in the retina following microglial miR155cKO suggest that an intricate regulation of pro- and anti-inflammatory cytokines is needed during the progression of retinal inflammation due to AD. Indeed, these data reveal a role for MGnD microglia in promoting retinal inflammation, which is suppressed by microglial miR-155cKO. It is noteworthy that a recent investigation found that isolated microglial cells from Galectin-3-KO mice exhibited decreased secretion of pro-inflammatory cytokines [8]. Therefore, it is conceivable that the decreased MGnD population achieved by miR-155 inhibition resulted in dampened pro-inflammatory microglial responses in the AD mouse retina, as observed in our study. An alternate contributing mechanism may be the protective phenotype of Cx3cr1 CreERT2 targeted perivascular macrophages. In accordance, a recent report demonstrated that Galectin-3 KO in the brains of Huntington’s disease model R6/2 mice significantly upregulated expression of IL-10 in striatal lysates [83]. Since increased expression of retinal IL-10 was observed in APP/PS1 mice with miR-155cKO, it is possible that the decrease in the Galectin-3^+^ MGnD microglia population in response to miR-155cKO intervention rescued the immunoregulatory activity of IL-10.

Our observation of global retinal proteomics signatures, with IPA analysis for canonical pathways, indicate upregulation of several PI3K-Akt signaling cascade pathways, including mTOR, EIF2, sirtuin and insulin secretion signaling, in APP/PS1 mice with miR-155 conditional inhibition. Indeed, mounting evidence establishes a protective role for the PI3K/Akt/mTOR pathway in retinal homeostasis. Specifically, a study using primary microglial cell cultures described an inhibitory effect of lithium against lipopolysaccharide (LPS)-induced microglial inflammation via activation of the PI3K/Akt/FoxO1 pathway [18]. Further, the PI3K/Akt signaling pathway was shown to mediate a protective 17β-estradiol effect of suppressing light-induced retinal neurodegeneration [93]. Our results are also consistent with those of a previous study in which miR-155 transfection in human nasopharyngeal cancer and human cervical cancer cells inhibited several components of PI3K-Akt-mTOR signaling, including RHEB, RICTOR, phosphorylated mTOR and Akt [91]. In fact, EIF2, sirtuin and insulin signaling pathways were all previously determined to be negative regulators of the pro-inflammatory NF-κB response [1, 77, 81]. Since we also detected upregulation of the aforementioned regulators along with reduced phosphorylation of the NF-κB p65 subunit in the retina of APP/PS1 mice with microglial miR-155 inhibition, we postulate that the anti-inflammatory effect of microglial miR-155cKO in the AD retina is partially due to activation of PI3K-Akt signaling cascades and decreased MGnD populations.

Upstream regulator analysis by IPA predicted inhibition of Spp1 (also referred to as OPN) and Cxcl-8 (also known as IL-8) in 8-month-old APP/PS1:miR-155cKO mice compared to APP/PS1 mice of the same age. Cxcl-8 is a chemokine that promotes leukostasis and inflammation in damaged tissues [6]. Spp1 is a cytokine that chemoattracts inflammatory cells and enhances activities of Th1 cytokines [55]. Therefore, this prediction further supports our findings of inhibited pro-inflammatory responses following miR-155cKO in APP/PS1 mice. Interestingly, our previous study identified essential roles of Spp1 in promoting Aβ phagocytosis and the anti-inflammatory phenotype in bone marrow-derived macrophages, while induction of Spp1 in cerebral-infiltrating macrophages, effective in Aβ clearance, was observed following glatiramer acetate immunization of APP_SWE_/PS1_dE9_ mice [74]. Notably, brain-resident microglial scRNAseq studies and meta-analysis of microglial genes identified Spp1 upregulation in the MGnD microglia of AD-model mice associated with restricting Aβ plaques [37, 47]. Here, we found Spp1 levels to be significantly reduced in the AD retina in response to microglial miR-155cKO. Yet, our immunostaining analysis indicates that retinal Spp1 is predominantly expressed by retinal ganglion cells and not microglia. Nevertheless, the roles of Spp1 and Cxcl-8 relating to retinal microglia should be further explored.

The protective effects of microglial miR-155 inhibition on retinal vascular integrity shine a new light on our previous identification of early and progressive retinal pericyte loss associated with increased vascular amyloidosis in mild cognitively impaired (MCI) and AD patients and APP/PS1 mice [78, 80]. In APP/PS1 mice, we previously determined an iBRB breakdown based on decreased expression of the key TJ molecules Claudin-1 and ZO-1 together with increased retinal vascular leakage [78]. Here, inhibition of microglial miR-155 protected impaired retinal vascular TJs in APP/PS1 mice. This is in agreement with findings of a previous study in which miR-155 inhibition in a human brain microvascular endothelial cell culture rescued expression of ZO-1 and Claudin-1 during oxygen–glucose deprivation [67]. We believe this protective effect of microglial miR-155cKO is due to decreased production of pro-inflammatory cytokines that are known to damage TJs, such as IL-6, IL-1β and TNF-α. Another possible mechanism is via miR-155cKO mediated loss of MGnD microglia, which was observed near retinal microvessels in the AD retina. Perivascular macrophages could also be affected by Cx3cr1-CreERT2–mediated miR-155 ablation and secrete fewer pro-inflammatory cytokines. Importantly, the TNF-α–activated NF-κB pathway was previously determined to downregulate TJs and induce endothelial permeability in a bovine retinal endothelial cell culture [4]. Since we detected decreased NF-κB p65 phosphorylation and TNF-α expression in AD retinas with miR-155cKO, it is likely that the protective effect of miR-155 targeting was partially through inhibition of TNF-α/NF-κB signaling.

It should be noted that due to the limited tissue size of mouse retina, we were unable to isolate retinal microglia in sufficient numbers for microglia-specific proteome analysis. Future studies are warranted for investigation of retinal Clec7a^+^ or Galectin-3^+^ microglia-specific molecular signatures in comparison to MGnD/DAM microglia in the brain. Nevertheless, our global proteome analysis provided mechanistic insights for the overall impact of microglial miR-155cKO in AD-model retina. An additional limitation is that due to the relatively small mice cohort available for MS analysis, we applied a 1.2-fold change cut-off when interpreting proteomics data. The long-term effect of miR-155cKO in older APP/PS1 mice at later disease stages, including in other AD models, should also be explored in the future.

## Conclusions

Taken together, our study revealed substantial populations of Clec7a^+^ and Galectin-3^+^ MGnD microglia associated with blood vessels in the retinas of AD-model mice and a reduced number of these MGnD microglia following targeted inhibition of miR-155. In this paper we describe the first observation of neurodegeneration-associated microglial phenotype in the retina of AD-model mice. Diverse microglial populations can be further explored for next-generation retinal microglial imaging to monitor AD progression and response to potential therapy. Our findings demonstrate the outstanding protective effects of microglial miR-155 inhibition: reduction in the number of retinal MGnD microglia, restoration of TJs in the iBRB, and decreased retinal vascular amyloidosis in murine models of AD. We identified that the mechanism involves, at least in part, an augmented PI3K-Akt signaling cascade and oxidative phosphorylation. Importantly, recent studies provide evidence for MGnD and homeostatic microglia in brains of AD patients and retinas of glaucoma patients and in healthy controls [8, 28, 39, 47, 58]. Our future study will further explore the roles of retinal MGnD vs. homeostatic microglia in relations to retinal pathology in AD patients. The translational potential of miR-155 inhibition as a treatment for AD should also be evaluated in animal models and human microglial cell cultures in the future. It is notable that the method of miR-155 inhibition was well established by using a locked nucleic acid (LNA) technology [10, 24]. Therefore, the feasibility of pharmacologically inhibiting miR-155 together with the protective effects of reducing MGnD by microglial miR-155cKO in the retina encourage the development of novel MGnD-targeted treatments for retinal vascular and inflammatory diseases.

## Supplementary Information

**Additional file1:** Supplementary Figures 1-9 and Tables 1-8.

## Data Availability

The data that support the findings of this study are available from the corresponding author, upon reasonable request.

## Abbreviations

miR: Micro-RNA
AD: Alzheimer’s disease
RNAseq: RNA sequencing
GWAS: Genome-wide association studies
CNS: Central nervous system
M0: Homeostatic microglia
TGFβ: Transforming growth factor beta
TREM2: Triggering receptor expressed on the myeloid cells 2
APOE: Apolipoprotein E
Aβ: Amyloid β
miR-155: MicroRNA-155
RICTOR: Rapamycin-insensitive companion of mammalian target of rapamycin
SMAD-1: SMAD family member 1
PI3K-Akt: Phosphoinositide 3-kinase-protein kinase B
TJ: Tight junctions
BBB: Blood–brain barrier
Cxcl-8: C-X-C motif chemokine ligand-8
Spp1: Secretion of phosphoprotein 1
APP/PS1: MiR155cKO: Cx3cr1^CreERT2^-miR155^fl/fl^-APP/PS1
cKO: Conditional knockout
Cx3cr1^CreERT2^: B6.129P2(Cg)-Cx3cr1^tm2.1(cre/ERT)Litt^/WganJ
Mir155^fl/fl^: C57BL/6-Mir155^tm1.1Ggard^/J
APP/PS1: APP_SWE_/PS1_L166P_
WT: Wild type
WTmiR155cKO: Cx3cr1^CreERT2^-miR155^fl/fl^-WT
IACUC: Institutional animal care and use committee
PFA: Paraformaldehyde
qRT-PCR: Quantitative reverse transcription polymerase chain reaction
HBSS: Hanks’ balanced salt solution
SEMs: Standard errors of the means
TBS: Tris-buffered saline
RT: Room temperature
PVDF: Polyvinylidene fluoride
TBST: Tris-buffered saline with Tween 20
BSA: Bovine serum albumin
MSD: Meso scale discovery
SDB-RPS: Styrene divinylbenzene‐reverse phase sulfonate
TMT: Tandem mass tag
MWD: Multi-wavelength detector
AGC: Automatic gain control
HCD: Higher energy collision-induced dissociation
NCE: Normalized collision energy
FAIMS: Field asymmetric ion mobility spectrometry
DV: Dispersion voltage
CVs: Compensation voltages
PSMs: Peptide-spectrum matches
FDR: False discovery rate
RA: Relative abundance
PCA: Principal component analysis
PANTHER: Protein annotation through evolutionary relationship
IPA: Ingenuity pathway analysis
IR: Immunoreactive
Tmem119: Transmembrane Protein 119
GCL: Ganglion cell layer
IPL: Inner plexiform layer
OPL: Outer plexiform layer
Iba1: Ionized calcium-binding adaptor protein-1
NF-κB: Nuclear factor kappa B
IL: Interleukin
TNF: Tumor necrosis factor
IFN-γ: Interferon-gamma
MS: Mass spectrometry
DEPs: Differentially expressed proteins
OXPHOS: Oxidative phosphorylation
iBRB: Inner blood-retinal barrier
MMP9: Matrix metallopeptidase 9
LPS: Lithium against lipopolysaccharide
MCI: Mild cognitively impaired
LNA: Locked nucleic acid
