# Supplementary material for: Regulating microglial miR-155 transcriptional phenotype alleviates Alzheimer’s-induced retinal vasculopathy by limiting Clec7a/Galectin-3+ neurodegenerative microglia
Source: Acta Neuropathol Commun. 2022 Sep 8;10:136. doi: 10.1186/s40478-022-01439-z (PMC9461176; doi:10.1186/s40478-022-01439-z)
Supplement: Supplementary file 1 — Additional file1: Supplementary Figures 1-9 and Tables 1-8. [file 40478_2022_1439_MOESM1_ESM.pdf]

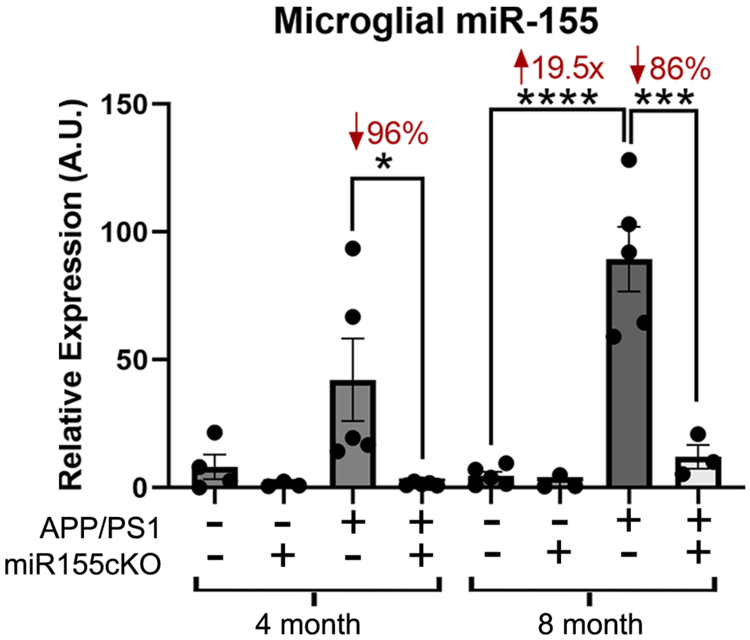

Supplementary figure 1. Quantitative reverse transcription polymerase chain reaction (qRT-PCR) analyses for expression of miR-155 in isolated microglia from mouse brains. *$p < 0.05$, ***$p < 0.001$, ****$p < 0.0001$, by one-way ANOVA with Tukey's post-hoc multiple comparison test. Fold changes and percentage decreases are shown in red.

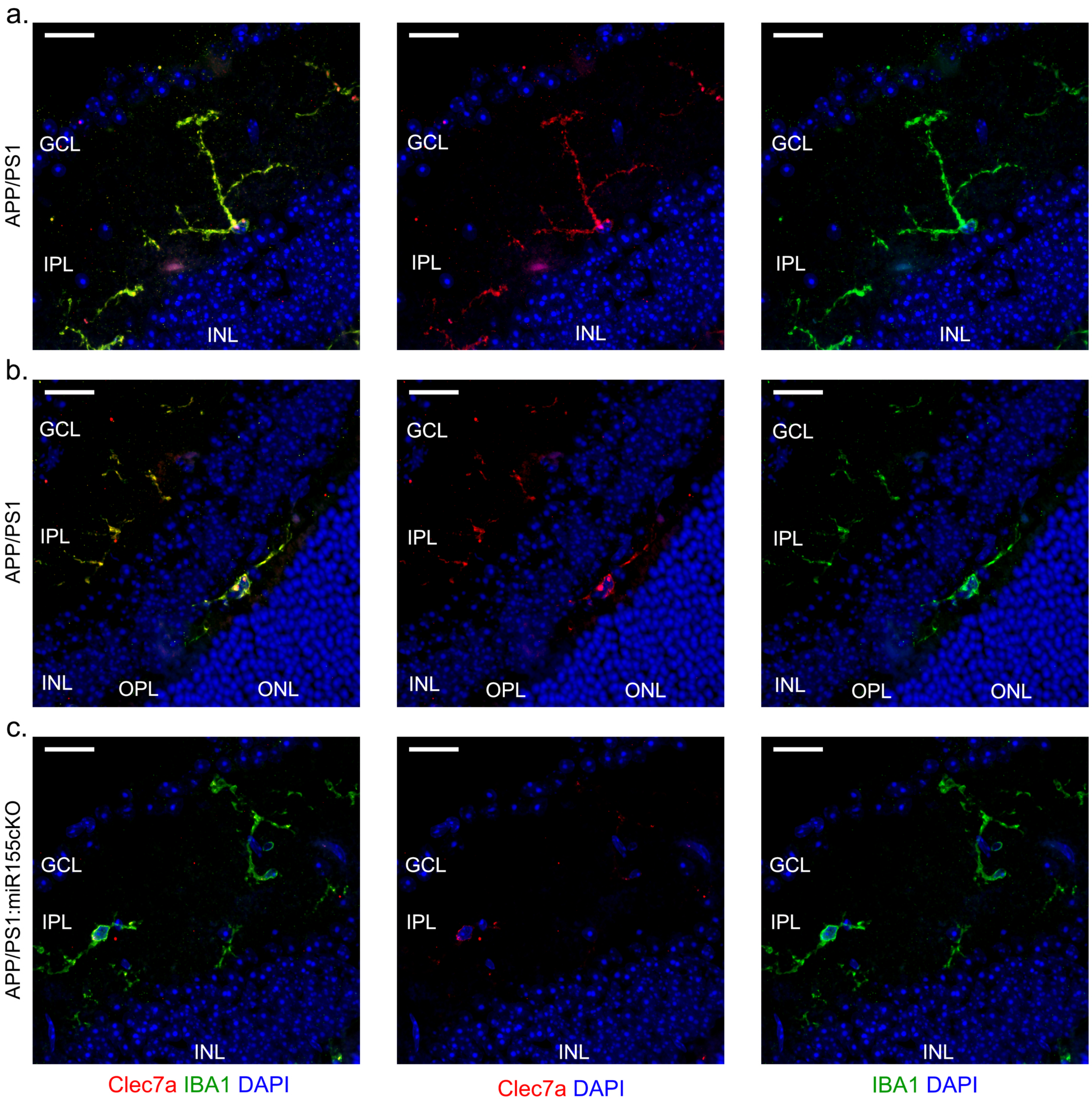

Supplementary figure 2. Retinal cross-section staining for Clec7a⁺ and Iba1⁺ microglia. **a-c.** Representative images of eye cross-section immunofluorescent staining for Clec7a⁺ microglia (red), Iba1⁺ microglia (green) and DAPI (blue) from **a-b.** APP/PS1 mice or **c.** APP/PS1:miR155cKO mice. Images were taken under 63x microscope objectives. Scale bars=10µm. GCL-ganglion cell layer. IPL-inner plexiform layer. INL-inner nuclear layer. OPL-outer plexiform layer. ONL-outer nuclear layer.

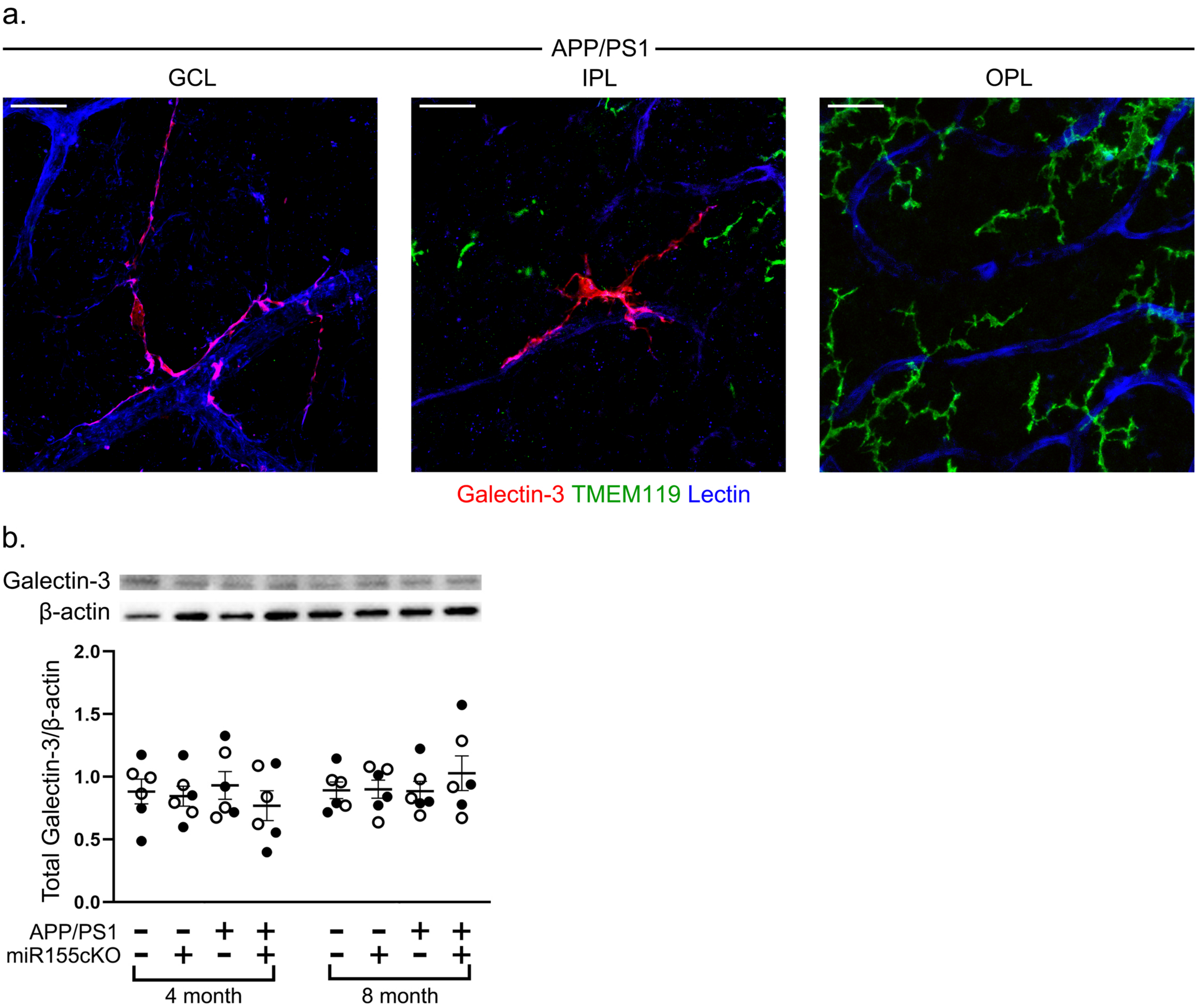

Supplementary figure 3. Supplementary data for Galectin-3⁺ microglia. **a.** Representative images of immunofluorescent staining for Galectin-3⁺ microglia (red), Tmem119⁺ microglia (green) and lectin for blood vessels (blue) on retinal flat-mounts from an APP/PS1 mouse. GCL-ganglion cell layer. IPL-inner plexiform layer. OPL-outer plexiform layer. Images were taken under 63x microscope objectives. Scale bars=10μm. **b.** Densitometric analysis of western-blotting protein bands of Galectin-3 normalized by β-actin control for retinal lysates from all experimental groups (total n=48, n=6 each group). Data from individual mouse (circles) as well as group means ± SEMs are shown. Black-filled circles represent males and clear circles represent females.

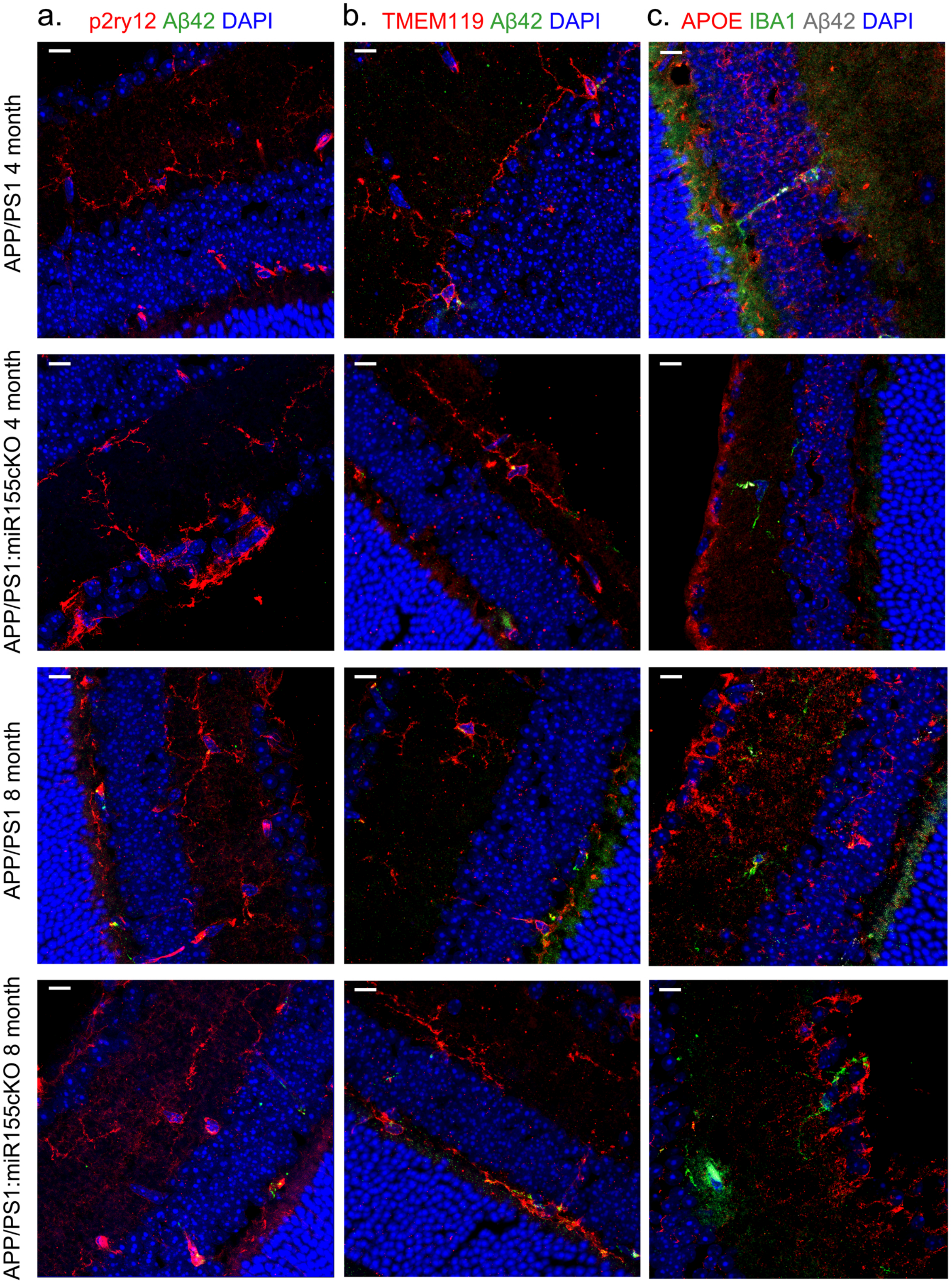

Supplementary figure 4. Representative images of P2ry12⁺, Tmem119⁺ and Apoe⁺ microglia in retinal cross-section. **a-c.** Representative images of immuno-fluorescent staining for a. P2ry12 (red)/Aβ42 (12F4, green)/DAPI (blue), **b.** Tmem119 (red)/Aβ42 (12F4, green)/DAPI (blue), and **c.** Apoe (red)/Iba1 (green)/Aβ42 (12F4, white)/DAPI (blue) on retinal cross-sections. Images were taken under 63x microscope objectives. Scale bars=10μm.

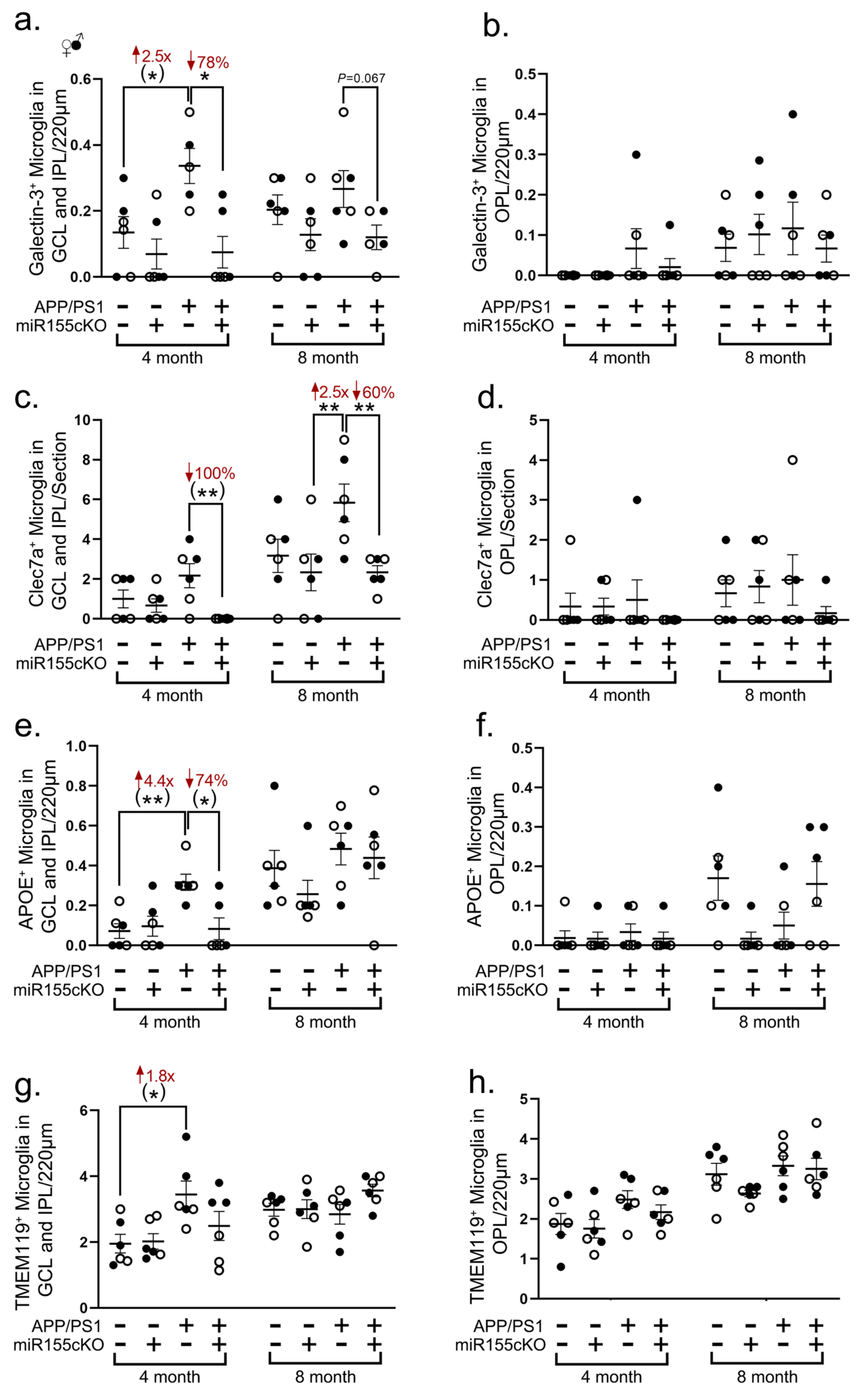

Supplementary figure 5. Extended manual counting of different phenotypes of microglia. **a-h.** Manual counting of **a-b.** Galectin-3⁺, **c-d.** Clec7a⁺, **e-f.** Apoe⁺, **g-h.** Tmem119⁺ microglia in retinal cross-sections from all experimental groups (total n=48, n=6 each group) in ganglion cell layers (GCL) and inner plexiform layers (IPL) (left row) versus outer plexiform layers (OPL) (right row). *p < 0.05, **p < 0.01, by three-way ANOVA with Tukey's post-hoc multiple comparison test. Two group statistical analysis was performed using an unpaired 2-tailed Student t-test and was shown in parentheses. Fold changes and percentage decreases are shown in red.

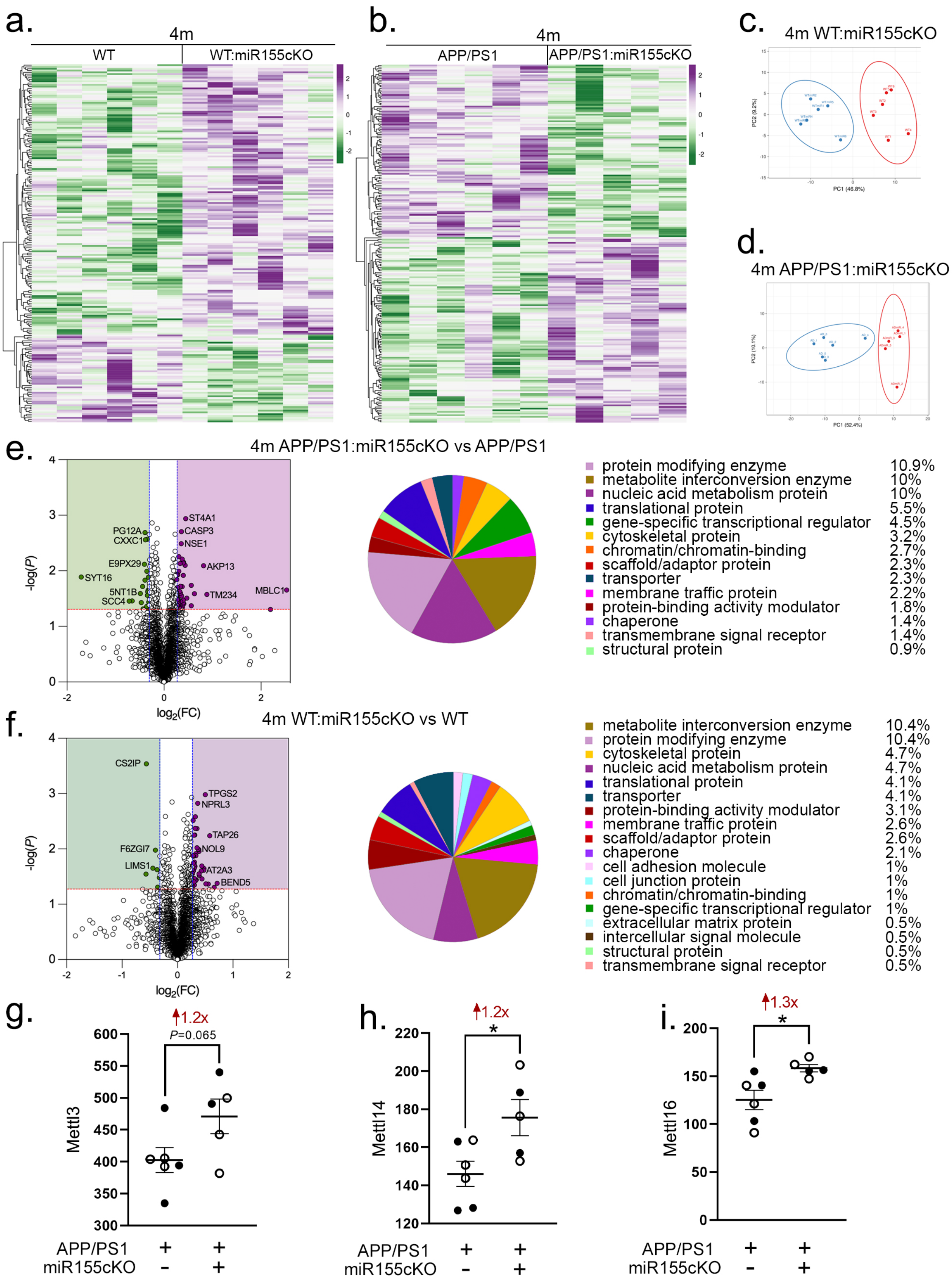

Supplementary figure 6. Retinal proteomic analysis for 4-month-old experimental groups. **a-b.** Detectable protein hierarchies displayed as heatmaps from **a.** 4-month-old WT:miR155cKO versus WT or **b.** 4-month-old APP/PS1:miR155cKO versus APP/PS1; upregulated proteins are shown in purple and downregulated proteins in green. **c-d.** Principal component analysis for **a.** and **b. e-f.** Volcano plots and Pie chart of PANTHER functional classification analysis for **a.** and **b. g-i.** Plotting of proteomics measurement of mettl proteins. *p < 0.05, by unpaired 2-tailed Student t-test. Fold changes are shown in red.

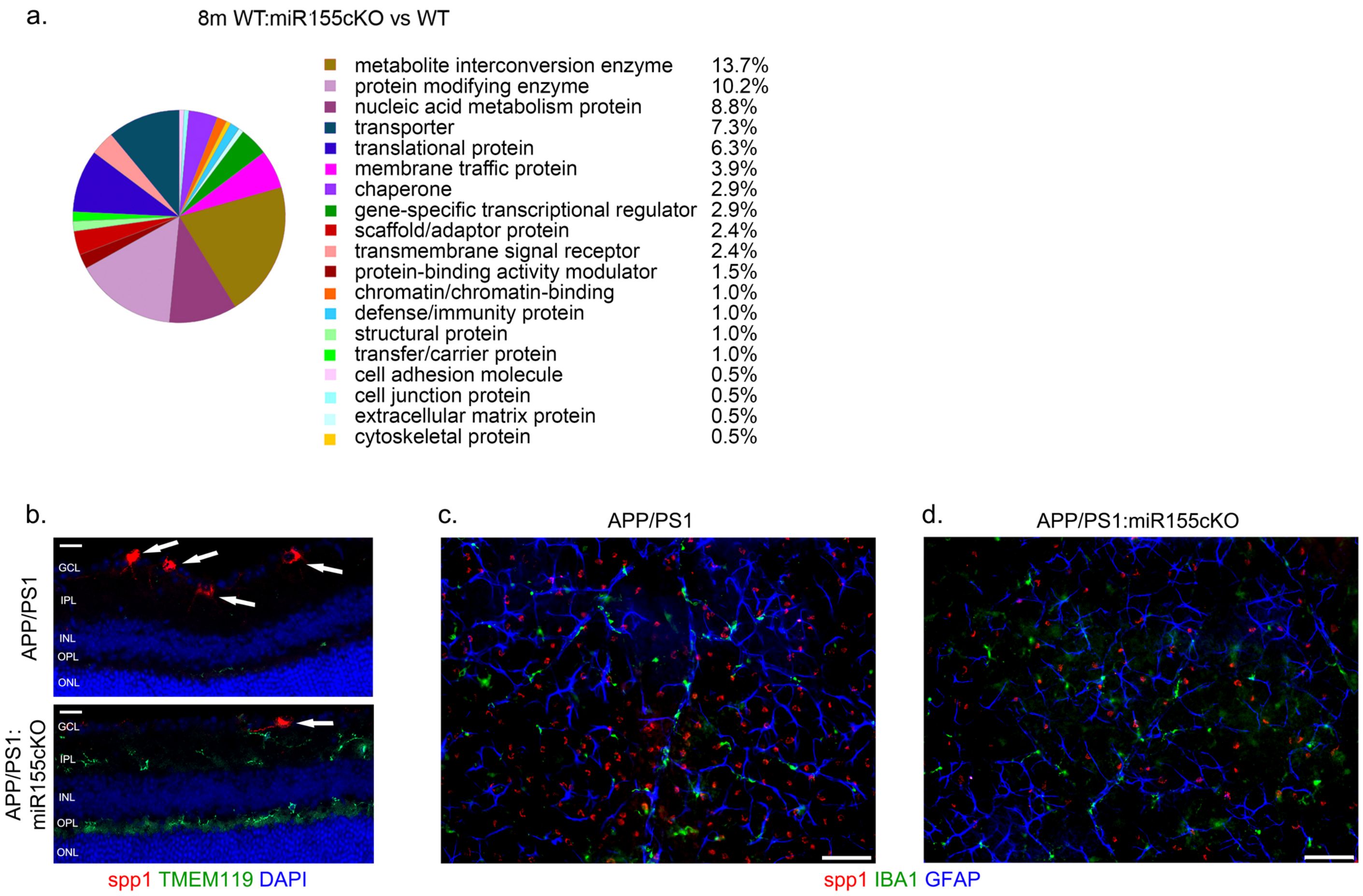

Supplementary figure 7. Extended proteomic data for figure 4. **a.** Pie chart of PANTHER functional classification analysis showing fraction and percentage of significant differentially expressed proteins (DEPs, up- or downregulated proteins) grouped by protein class category based on 8-month-old WT:miR155cKO versus WT. **b.** Representative images of immunofluorescent staining for Spp1 (red)/Tmem119 (green)/DAPI (blue) on retinal cross-sections. Images were taken under 20x microscope objectives. Scale bars=10μm. **c-d.** Representative images of immunofluorescent staining for Spp1 (red), Iba1 (green) and Gfap (blue) on retinal flat-mounts from **c.** APP/PS1 mouse or **d.** APP/PS1:miR155cKO mouse. Images were taken under 10x microscope objectives. Scale bars=10μm.

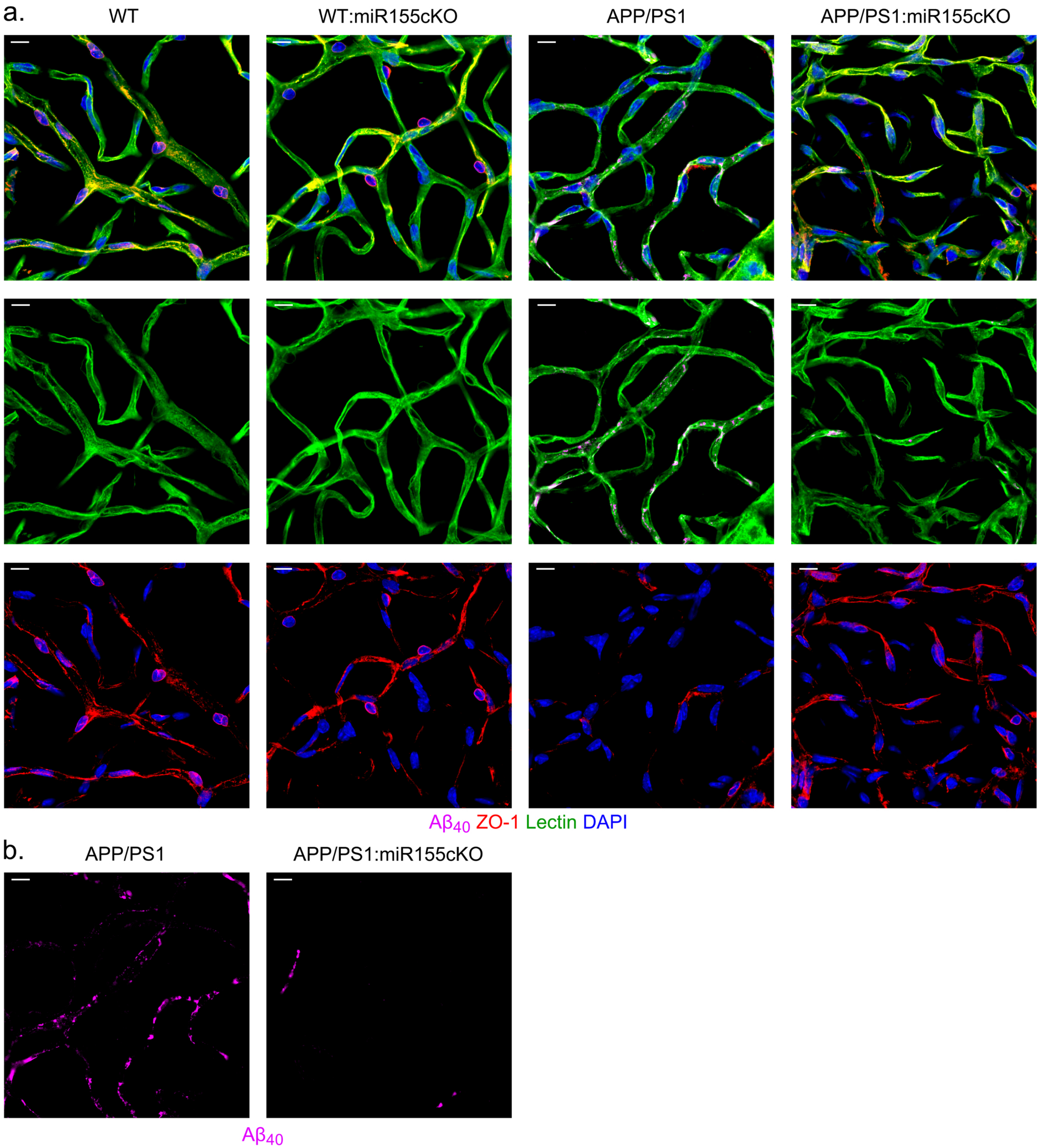

Supplementary figure 8. Extensive images and data analysis for retinal vascular ZO-1 and Aβ40. **a.** Representative images of immunofluorescent staining for 11A50-B10 of Aβ40 (magenta), ZO-1 (red), lectin for blood vessels (green) and DAPI (blue) on isolate retinal blood vessels from all four genotypes of WT, WT:miR155cKO, APP/PS1, APP/PS1:miR155cKO at 8-month-old mouse age. Images were taken under 63x microscope objectives. Scale bars=10μm. **b.** Representative images only showing differences of retinal vascular Aβ40.

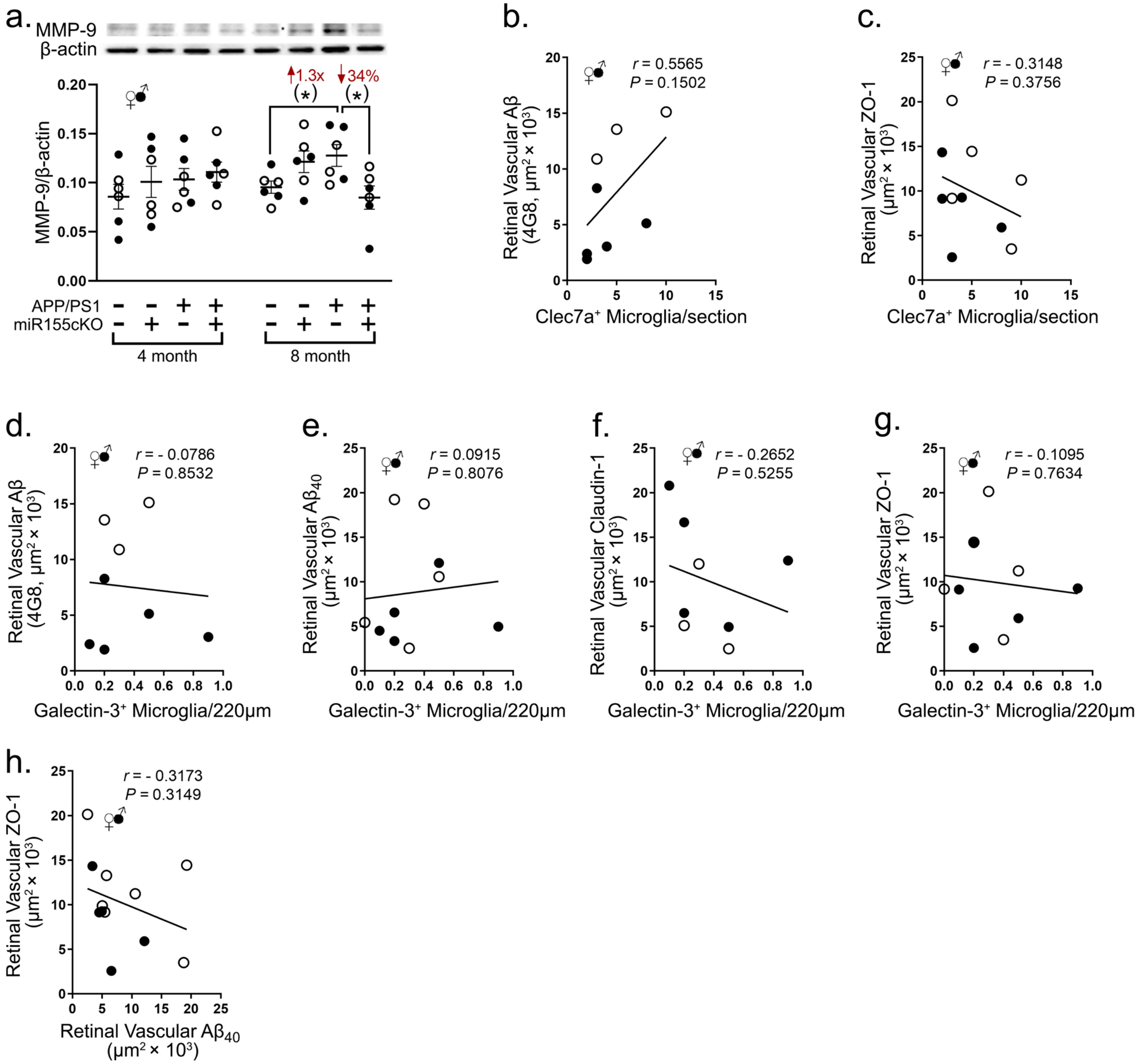

Supplementary figure 9. Supplementary data analyses for figure 5. **a.** Densitometric analysis of western-blotting protein bands of Mmp-9 normalized by β-actin control for retinal lysates from all experimental groups (total n=48, n=6 each group). **b-c.** Pearson's coefficient (r) correlation between retinal Clec7a⁺ microglia versus **b.** retinal vascular A β- immunoreactive area (IR) or **c.** retinal vascular ZO-1-IR. **d-g.** Pearson's coefficient (r) correlation between Galectin-3⁺ microglia versus **d.** retinal vascular Aβ-IR, **e.** retinal vascular Aβ40-IR, **f.** retinal vascular claudin-1-IR, or **g.** retinal vascular ZO-1-IR. **h.** Pearson's coefficient (r) correlation between retinal vascular ZO-1-IR versus retinal vascular Aβ40-IR. Data from individual mouse (circles) as well as group means ± SEMs are shown. Black-filled circles represent males and clear circles represent females. *$p < 0.05$, by three-way ANOVA with Tukey's post-hoc multiple comparison test. Two group statistical analysis was performed using an unpaired 2-tailed Student t-test and is shown in parentheses. Fold changes and percentage decreases are shown in red.

Table S1. Mass spectrometry data showing significantly up-regulated proteins in 8-month-old APP/PS1:miR155cKO compared to APP/PS1 control group.

Accession	Symbol	Description	APP/PS1:miR155cKO (Mean)	APP/PS1 (Mean)	FC	p-value
O54828-2	RGS9	Regulator of G-protein signaling 9-binding protein	8851.83	5060.25	1.75	0.04294
Q80XC6	NRDE2	Nuclear exosome regulator NRDE2	37.74	23.08	1.64	0.03696
Q61012	GNGT1	Guanine nucleotide-binding protein G(T) subunit gamma-T1	14957.02	9400.30	1.59	0.04072
Q8BMD6	TM260	Transmembrane protein 260	366.84	243.61	1.51	0.01259
Q8BYK4	RDH12	Retinol dehydrogenase 12	4665.08	3182.26	1.47	0.00643
Q3U4N2	ICMT	Protein-S-isoprenylcysteine O-methyltransferase	197.80	135.79	1.46	0.02107
G3X8W7	CDC23	Cell division cycle protein 23 homolog	629.60	434.31	1.45	0.03146
Q8R2K4	TAF6L	TAF6-like RNA polymerase II p300/CBP-associated factor-associated factor 65 kDa subunit	71.28	49.22	1.45	0.00532
Q8K245	UVRAG	UV radiation resistance-associated protein	46.84	32.44	1.44	0.01872
P61358	RPL27	60S ribosomal protein L27	3032.77	2122.52	1.43	0.04167
Q8BM55	TMEM214	Transmembrane protein 214	82.86	58.21	1.42	0.04954
Q8BFV2	PCID2	PCI domain-containing protein 2	152.91	108.80	1.41	0.00188
Q5XJY4	PARL	Presenilins-associated rhomboid-like protein, mitochondrial	196.30	141.04	1.39	0.03213
Q99LC3	NDUFA10	NADH dehydrogenase [ubiquinone] 1 alpha subcomplex subunit 10, mitochondrial	2696.30	1937.99	1.39	0.01325
G3X8Y3	NAA15	N-alpha-acetyltransferase 15, NatA auxiliary subunit	1572.64	1133.16	1.39	0.03190
Q6ZQ38	CAND1	Cullin-associated NEDD8-dissociated protein 1	9306.35	6710.49	1.39	0.00546
Q9CPZ8	CMC1	COX assembly mitochondrial protein homolog	118.74	86.49	1.37	0.01423
Q9CQA1	TRAPPC5	Trafficking protein particle complex subunit 5	225.94	166.91	1.35	0.00513
Q9D0P0	EBPL	Emopamil-binding protein-like	664.75	491.31	1.35	0.02165
Q8R1B4	EIF3C	Eukaryotic translation initiation factor 3 subunit C	3047.71	2265.08	1.35	0.02359
Q3TYD6	LMTK2	Serine/threonine-protein kinase LMTK2	39.45	29.46	1.34	0.00454
Q9DC69	NDUFA9	NADH dehydrogenase [ubiquinone] 1 alpha subcomplex subunit 9, mitochondrial	8303.90	6246.72	1.33	0.03466
Q80U95	UBE3C	Ubiquitin-protein ligase E3C	63.19	48.02	1.32	0.03905
P62702	RPS4X	40S ribosomal protein S4, X isoform	5196.95	3951.23	1.32	0.04156
Q8CDM8	F16B1	Protein FAM160B1	60.83	46.33	1.31	0.04588
P12970	RPL7A	60S ribosomal protein L7a	4934.32	3778.63	1.31	0.04812

P62751	RPL23A	60S ribosomal protein L23a	3566.97	2738.22	1.30	0.02698
O70152	DPM1	Dolichol-phosphate mannosyltransferase subunit 1	794.79	611.13	1.30	0.01654
O89112	LANCL1	Glutathione S-transferase LANCL1	400.42	308.60	1.30	0.04268
Q7TPD0	INTS3	Integrator complex subunit 3	1017.51	784.33	1.30	0.01690
Q8BWZ3	NAA25	N-alpha-acetyltransferase 25, NatB auxiliary subunit	431.52	334.86	1.29	0.01678
Q3UR70	TGFA1	Transforming growth factor-beta receptor-associated protein 1	67.11	52.29	1.28	0.04711
Q8R0X7	SGPL1	Sphingosine-1-phosphate lyase 1	198.49	155.32	1.28	0.03852
Q9WUD8	FAIM1	Fas apoptotic inhibitory molecule 1	1715.47	1349.28	1.27	0.03736
O70299	MB211	Putative nucleotidyltransferase MAB21L1	553.40	436.13	1.27	0.02553
Q9D6D0	SLC25A27	Solute carrier family 25, member 27	273.69	215.91	1.27	0.02669
P62843	RPS15	40S ribosomal protein S15	893.84	715.94	1.25	0.04849
Q91YM2	RHG35	Rho GTPase-activating protein 35	160.72	128.76	1.25	0.04293
Q9D5V5	CUL5	Cullin-5	2704.15	2168.47	1.25	0.03624
Q9CZV8-3	FXL20	Isoform 3 of F-box/LRR-repeat protein 20	338.33	271.65	1.25	0.04389
Q8QZY1	EIF3L	Eukaryotic translation initiation factor 3 subunit L	1963.54	1580.83	1.24	0.01515
Q64511	TOP2B	DNA topoisomerase 2-beta	15998.51	12882.18	1.24	0.03723
Q6A026	PDS5A	Sister chromatid cohesion protein PDS5 homolog A	370.11	300.00	1.23	0.00727
P61222	ABCE1	ATP-binding cassette sub-family E member 1	1422.15	1154.09	1.23	0.04248
Q9CQZ5	NDUA6	NADH dehydrogenase [ubiquinone] 1 alpha subcomplex subunit 6	2672.37	2173.34	1.23	0.01228
Q8BVY0	RL1D1	Ribosomal L1 domain-containing protein 1	1488.27	1212.46	1.23	0.02636
Q9D5V6	SYAP1	Synapse-associated protein 1	208.33	170.61	1.22	0.02340
Q8BM39	PRP18	Pre-mRNA-splicing factor 18	117.18	96.09	1.22	0.00834
Q9DB50	AP1S2	AP-1 complex subunit sigma-2	324.84	266.45	1.22	0.02109
O08547	SC22B	Vesicle-trafficking protein SEC22b	3138.28	2577.43	1.22	0.02976
A0A0J9YUD5	TMLH	Nucleoporin 205	923.73	758.79	1.22	0.03733
Q8C0C7	SYFA	Phenylalanine--tRNA ligase alpha subunit	2675.69	2200.27	1.22	0.03388
P62245	RS15A	40S ribosomal protein S15a	3190.47	2634.39	1.21	0.02347
Q99NB9	SF3B1	Splicing factor 3B subunit 1	6316.50	5215.61	1.21	0.02960
Q9D517	PLCC	1-acyl-sn-glycerol-3-phosphate acyltransferase gamma	6490.16	5388.21	1.20	0.02989
Q9JHU4	DYHC1	Cytoplasmic dynein 1 heavy chain 1	44521.14	36970.68	1.20	0.03566

APP/PS1, $APP_{SWE}/PS1_{L166P}$ double transgenic mouse model of Alzheimer's disease (AD); APP/PS1:miR155cKO, $Cx3cr1^{CreERT2}$-$miR155^{fl/fl}$-APP/PS1 mice; FC, fold-change (FC>1.2 included); *P*<0.05

Table S2. Mass spectrometry data showing significantly down-regulated proteins in 8-month-old APP/PS1:miR155cKO compared to APP/PS1 control group.

Accession	Symbol	Description	APP/PS1:miR155cKO (Mean)	APP/PS1 (Mean)	FC	p-value
P28481	CO2A1	Collagen alpha-1(II) chain	44.12	76.28	-1.73	0.00601
Q91WE6	CDKAL	Threonylcarbamoyladenosine tRNA methylthiotransferase	16.53	27.70	-1.68	0.01085
Q99PH1	LRRC4	Leucine-rich repeat-containing protein 4	86.53	135.16	-1.56	0.00702
Q6P6M7	SPCS	O-phosphoseryl-tRNA(Sec) selenium transferase	28.43	44.01	-1.55	0.04470
Q3U0L2	AN33B	Ankyrin repeat domain-containing protein 33B	485.82	733.96	-1.51	0.02989
P49935	CATH	Pro-cathepsin H	411.93	619.70	-1.50	0.00715
Q8VC52	RBPS2	RNA-binding protein with multiple splicing 2	258.17	387.33	-1.50	0.02890
Q3UHH1	ZSWM8	Zinc finger SWIM domain-containing protein 8	43.33	63.59	-1.47	0.02009
Q6WKZ8	UBR2	E3 ubiquitin-protein ligase UBR2	74.67	108.25	-1.45	0.00218
Q8BUV6	LSM11	U7 snRNA-associated Sm-like protein LSm11	18.25	26.41	-1.45	0.03990
Q8BG47	RN152	E3 ubiquitin-protein ligase RNF152	42.95	62.00	-1.44	0.03070
Q9WV91	FPRP	Prostaglandin F2 receptor negative regulator	73.12	105.16	-1.44	0.02023
P97490	ADCY8	Adenylate cyclase type 8	411.72	590.54	-1.43	0.02513
A2A699	F1712	Protein FAM171A2	82.29	117.94	-1.43	0.02946
O55026	ENTP2	Ectonucleoside triphosphate diphosphohydrolase 2	213.30	305.52	-1.43	0.03235
P51569	AGAL	Alpha-galactosidase A	77.16	110.33	-1.43	0.02706
Q61627	GRID1	Glutamate receptor ionotropic, delta-1	44.36	62.51	-1.41	0.04852
Q9CQP3	CHCH5	Coiled-coil-helix-coiled-coil-helix domain-containing protein 5	79.75	112.16	-1.41	0.02656
Q761V0	SC6A5	Sodium- and chloride-dependent glycine transporter 2	111.57	154.10	-1.38	0.02882
Q9CQN7	RM41	39S ribosomal protein L41, mitochondrial	371.61	506.55	-1.36	0.02811
Q6PGH2	JUPI2	Jupiter microtubule associated homolog 2	144.91	196.52	-1.36	0.01978
Q61247	A2AP	Alpha-2-antiplasmin	143.29	192.69	-1.34	0.01873
Q8VCQ3	NRBF2	Nuclear receptor-binding factor 2	35.26	47.36	-1.34	0.00214
P43274	H14	Histone H1.4	15743.48	21052.68	-1.34	0.04002
Q9D937	CK098	Uncharacterized protein C11orf98 homolog	663.51	871.72	-1.31	0.02661
P01864	GCAB	Ig gamma-2A chain C region secreted form	143.83	188.42	-1.31	0.01078
Q6KAU4	MB12B	Multivesicular body subunit 12B	48.45	63.30	-1.31	0.04273

Q62470-2	ITA3	Isoform 2 of Integrin alpha-3	209.21	272.95	-1.30	0.01935
Q60841	RELN	Reelin	165.11	215.32	-1.30	0.01426
Q91XF0	PNPO	Pyridoxine-5'-phosphate oxidase	133.64	173.68	-1.30	0.03187
Q08481	PECA1	Platelet endothelial cell adhesion molecule	38.24	49.25	-1.29	0.04796
P55200	KMT2A	Histone-lysine N-methyltransferase 2A	96.64	123.98	-1.28	0.00616
Q8VEH2	Q8VEH2	CDKN1A-interacting zinc finger protein 1	159.10	202.61	-1.27	0.04636
Q9QZB0	RGS17	Regulator of G-protein signaling 17	99.50	126.50	-1.27	0.03956
P50171-2	DHB8	Isoform Long of Estradiol 17-beta-dehydrogenase 8	895.48	1134.37	-1.27	0.01952
Q8CIF4	BTD	Biotinidase	64.72	81.80	-1.26	0.00647
Q9CY73	RM44	39S ribosomal protein L44, mitochondrial	117.17	147.76	-1.26	0.03149
Q8R205	ZC3HA	Zinc finger CCCH domain-containing protein 10	53.96	67.72	-1.26	0.03078

APP/PS1, $APP_{SWE}/PS1_{L166P}$ double transgenic mouse model of Alzheimer's disease (AD); APP/PS1:miR155cKO, $Cx3cr1^{CreERT2}$-$miR155^{fl/fl}$-APP/PS1 mice; FC, fold-change (FC>1.2 included); $P<0.05$

Table S3. Mass spectrometry data showing significantly up-regulated proteins in 8-month-old WT:miR155cKO compared to WT control group.

Accession	Symbol	Description	WT:miR155cKO (Mean)	WT (Mean)	FC	p-value
Q8BVZ5	IL33	Interleukin-33	38.03	22.04	1.73	0.00995
Q9CPR5	RM15	39S ribosomal protein L15, mitochondrial	228.26	147.36	1.55	0.00572
Q99LH4	ZN672	Zinc finger protein 672	79.05	55.09	1.43	0.01621
Q7TPD0	INT3	Integrator complex subunit 3	1183.38	832.01	1.42	0.01156
P61358	RL27	60S ribosomal protein L27	2821.01	2016.36	1.40	0.02475
Q91YU8	SSF1	Suppressor of SWI4 1 homolog	57.93	43.05	1.35	0.02264
Q6A0A2	LAR4B	La-related protein 4B	279.61	210.69	1.33	0.04240
Q64511	TOP2B	DNA topoisomerase 2-beta	15693.44	11904.76	1.32	0.04752
F6Q1V8	F6Q1V8	FRY-like transcription coactivator (Fragment)	273.80	208.95	1.31	0.02230
B2RX14	TUT4	Terminal uridylyltransferase 4	26.68	20.78	1.28	0.04087
Q8BX57	PXK	PX domain-containing protein kinase-like protein	72.88	57.73	1.26	0.00521
Q8BVY0	RL1D1	Ribosomal L1 domain-containing protein 1	1474.68	1177.59	1.25	0.02007
Q8K205	Q8K205	Processing of 1, ribonuclease P/MRP family, (S. cerevisiae)	218.75	175.36	1.25	0.00881
Q9JHW4	SELB	Selenocysteine-specific elongation factor	157.35	126.42	1.24	0.01830
Q5XJF6	Q5XJF6	Ribosomal protein	2132.84	1715.17	1.24	0.03475
Q689Z5-2	SBNO1	Isoform 2 of Protein strawberry notch homolog 1	162.27	131.41	1.23	0.01316
P56960	EXOSX	Exosome component 10	1275.44	1037.48	1.23	0.01788
Q9EQC5	SCYL1	N-terminal kinase-like protein	143.04	116.46	1.23	0.00662
Q0VGB7	PP4R2	Serine/threonine-protein phosphatase 4 regulatory subunit 2	1133.28	925.46	1.22	0.02356
P14131	RS16	40S ribosomal protein S16	3654.43	2987.11	1.22	0.03376
Q6KCD5	NIPBL	Nipped-B-like protein	470.37	384.57	1.22	0.04458
P62702	RS4X	40S ribosomal protein S4, X isoform	5279.36	4321.45	1.22	0.03280
Q9QZW9	MNX1	Motor neuron and pancreas homeobox protein 1	515.65	422.11	1.22	0.01288
Q91W96	APC4	Anaphase-promoting complex subunit 4	52.89	43.65	1.21	0.03643
B7ZMP1	XPP3	Xaa-Pro aminopeptidase 3	207.61	172.86	1.20	0.00990
Q9D787	PPIL2	RING-type E3 ubiquitin-protein ligase PPIL2	221.11	184.11	1.20	0.00512
Q0VBD0	ITB8	Integrin beta-8 OS=Mus musculus OX=10090 GN=Itgb8 PE=1 SV=1	437.17	365.22	1.20	0.03026

WT,wild type mice; WT:miR155cKO, Cx3cr1^{CreERT2}-miR155$^{fl/fl}$-WT mice; FC, fold-change (FC>1.2 included); $P<0.05$

Table S4. Mass spectrometry data showing significantly down-regulated proteins in 8-month-old WT:miR155cKO compared to WT control group.

Accession	Symbol	Description	WT:miR155cKO (Mean)	WT (Mean)	FC	p-value
Q6P5D4	CP135	Centrosomal protein of 135 kDa	83.81	144.63	-1.73	0.03285
Q69ZS0	PZRN3	####E3 ubiquitin-protein ligase PDZRN3	41.59	65.86	-1.58	0.03238
Q9WV35	ABEC2	C->U-editing enzyme APOBEC-2	148.64	233.48	-1.57	0.04232
E9Q8Q6	E9Q8Q6	Coiled-coil domain-containing 141	80.02	121.60	-1.52	0.02116
Q61733	RT31	28S ribosomal protein S31, mitochondrial	64.82	94.61	-1.46	0.02353
A9Z1V5	VW5B1	von Willebrand factor A domain-containing protein 5B1	267.73	381.46	-1.42	0.00985
Q80SY4	MIB1	E3 ubiquitin-protein ligase MIB1	136.75	189.77	-1.39	0.04529
Q8VHW4	CCG5	Voltage-dependent calcium channel gamma-5 subunit	217.67	301.66	-1.39	0.00429
Q9R1V7-3	ADA23	Isoform Gamma of Disintegrin and metalloproteinase domain-containing protein 23	16.78	23.20	-1.38	0.03426
Q91WE6	CDKAL	Threonylcarbamoyladenosine tRNA methylthiotransferase	16.13	21.26	-1.32	0.00727
O70338	RNH1	Ribonuclease H1	15.77	20.68	-1.31	0.04195
P47743	GRM8	Metabotropic glutamate receptor 8	66.13	86.48	-1.31	0.02860
P28798	GRN	Progranulin	266.18	346.36	-1.30	0.04441
O08688	CAN5	Calpain-5	45.89	59.29	-1.29	0.00300
Q923D4	SF3B5	Splicing factor 3B subunit 5	295.49	378.23	-1.28	0.02634
P06797	CATL1	Cathepsin L1	93.01	118.68	-1.28	0.01101
Q61069	USF1	Upstream stimulatory factor 1	162.98	207.45	-1.27	0.00629
P40237	CD82	CD82 antigen	253.29	321.42	-1.27	0.02717
Q9DCN1	NUD12	Peroxisomal NADH pyrophosphatase NUDT12	38.06	48.26	-1.27	0.01094
Q9EPR2	PG12A	Group XIIA secretory phospholipase A2	57.92	73.33	-1.27	0.02601
Q6NVE9	PPTC7	Protein phosphatase PTC7 homolog	57.50	72.65	-1.26	0.04822
Q8BGX3	LRTM2	Leucine-rich repeat and transmembrane domain-containing protein 2	92.22	116.28	-1.26	0.03734
Q78JW9	UBFD1	Ubiquitin domain-containing protein UBFD1	123.38	155.51	-1.26	0.00201

WT,wild type mice; WT:miR155cKO, Cx3cr1^{CreERT2}-miR155$^{fl/fl}$-WT mice; FC, fold-change (FC>1.2 included); *P*<0.05

Table S5. Mass spectrometry data showing significantly up-regulated proteins in 4-month-old APP/PS1:miR155cKO compared to APP/PS1 control group.

Accession	Symbol	Description	APP/PS1:miR155cKO (Mean)	APP/PS1 (Mean)	FC	p-value
Q8BWY4	MBLC1	Metallo-beta-lactamase domain-containing protein 1	442.86	76.73	5.77	0.02211
Q9R269	PEPL	Periplakin	198.12	43.20	4.59	0.04968
Q8R1E7	TM234	Transmembrane protein 234	29.70	16.10	1.84	0.02661
E9Q394	AKP13	A-kinase anchor protein 13	102.40	58.26	1.76	0.00813
Q9D0B6	PBDC1	Protein PBDC1	84.14	54.41	1.55	0.02585
Q14B01	Q14B01	Ring finger protein 113A2	52.79	35.69	1.48	0.01826
P26350	PTMA	Prothymosin alpha	558.54	378.10	1.48	0.04257
Q5U901	CBPA6	Carboxypeptidase A6	848.75	611.95	1.39	0.03097
P63046	ST4A1	Sulfotransferase 4A1	73.67	54.01	1.36	0.00116
Q69Z89	RADIL	Ras-associating and dilute domain-containing protein	116.69	86.30	1.35	0.00796
Q8C5R2	PRSR2	Proline and serine-rich protein 2	42.08	31.66	1.33	0.03895
Q7TPD0	INT3	Integrator complex subunit 3	1231.32	930.44	1.32	0.00704
Q3TMH2	SCRN3	Secernin-3	438.51	332.43	1.32	0.01205
Q8BGQ1	SPE39	Spermatogenesis-defective protein 39 homolog	66.15	50.16	1.32	0.03753
Q9Z2L7	CRLF3	Cytokine receptor-like factor 3	376.73	285.88	1.32	0.00720
Q8VCA8	SCRN2	Secernin-2	385.39	292.63	1.32	0.03366
A0A087WPA0	A0A087WPA0	LBH domain-containing 1	348.98	267.56	1.30	0.04470
Q71FD5	ZNRF2	E3 ubiquitin-protein ligase ZNRF2	66.46	51.21	1.30	0.01927
Q8K2X3	STN1	CST complex subunit STN1	146.67	113.09	1.30	0.00615
O88848-2	ARL6	Isoform 2 of ADP-ribosylation factor-like protein 6	359.40	279.05	1.29	0.02837
P70677	CASP3	Caspase-3	216.81	169.01	1.28	0.00197
Q9D720	NSE1	Non-structural maintenance of chromosomes element 1 homolog	34.46	26.97	1.28	0.00325
Q99PM3	TF2AA	Transcription initiation factor IIA subunit 1	203.78	159.73	1.28	0.04603
Q91WU5	AS3MT	Arsenite methyltransferase	615.22	485.20	1.27	0.03591
Q9DBX2	PHLP	Phosducin-like protein	2513.16	1983.54	1.27	0.02516

Q9CQG2	MET16	RNA N6-adenosine-methyltransferase METTL16	158.38	125.25	1.26	0.01893
Q9JMA2	TGT	Queuine tRNA-ribosyltransferase catalytic subunit 1	50.92	40.47	1.26	0.00739
Q9D0U6	MAF1	Repressor of RNA polymerase III transcription MAF1 homolog	47.41	37.82	1.25	0.02360
Q8VCN5	CGL	Cystathionine gamma-lyase	64.71	51.76	1.25	0.04127
P56375	ACYP2	Acylphosphatase-2	968.73	777.72	1.25	0.04660
Q8CAY6	THIC	Acetyl-CoA acetyltransferase, cytosolic	1231.47	995.93	1.24	0.01023
Q69ZC8	GPAM1	GPALPP motifs-containing protein 1	103.69	83.90	1.24	0.00555
Q8BH58	TIPRL	TIP41-like protein	408.89	332.74	1.23	0.03517
F6U3S2	F6U3S2	Major facilitator superfamily domain-containing protein 6 (Fragment)	24.60	20.06	1.23	0.04139
Q9Z0E6	GBP2	Guanylate-binding protein 2	208.60	170.27	1.23	0.03265
P13439	UMPS	Uridine 5'-monophosphate synthase	458.14	374.54	1.22	0.04080
Q9JKX6	NUDT5	ADP-sugar pyrophosphatase	195.90	160.53	1.22	0.03898
Q9CXU9	EIF1B	Eukaryotic translation initiation factor 1b	121.63	99.94	1.22	0.01180
Q8CHX7	RFTN2	Raftlin-2	124.32	102.53	1.21	0.02000
Q5SYD0	MYO1D	Unconventional myosin-Id	64.96	53.58	1.21	0.03288
Q8BP27	SFR1	Swi5-dependent recombination DNA repair protein 1 homolog	317.44	262.59	1.21	0.03327
Q9D967	MGDP1	Magnesium-dependent phosphatase 1	464.61	385.11	1.21	0.04149
Q3UIK4	MET14	N6-adenosine-methyltransferase non-catalytic subunit	175.64	146.06	1.20	0.02770
A2RTH5	A2RTH5	Leucine carboxyl methyltransferase 1	162.44	135.15	1.20	0.01194
Q8C8T7	ELFN1	Protein ELFN1	346.67	288.53	1.20	0.01441
O88271	CFDP1	Craniofacial development protein 1	3406.87	2837.86	1.20	0.02677

APP/PS1, $APP_{SWE}/PS1_{L166P}$ double transgenic mouse model of Alzheimer's disease (AD); APP/PS1:miR155cKO, $Cx3cr1^{CreERT2}$-$miR155^{fl/fl}$-APP/PS1 mice; FC, fold-change (FC>1.2 included); *P*<0.05

Table S6. Mass spectrometry data showing significantly down-regulated proteins in 4-month-old APP/PS1:miR155cKO compared to APP/PS1 control group.

Accession	Symbol	Description	APP/PS1:miR155cKO (Mean)	APP/PS1 (Mean)	FC	p-value
Q7TN83	SYT16	Synaptotagmin-16	184.40	602.72	-3.27	0.01298
Q9D2X5	SCC4	MAU2 chromatid cohesion factor homolog	85.09	139.83	-1.64	0.03511
P18608	HMGN1	Non-histone chromosomal protein HMG-14	8915.34	14037.37	-1.57	0.03493
Q91YE9	5NT1B	Cytosolic 5'-nucleotidase 1B	199.83	280.03	-1.40	0.02562
Q9DB42	ZN593	Zinc finger protein 593	111.88	154.71	-1.38	0.03722
G5E8C2	G5E8C2	Kelch repeat and BTB (POZ) domain-containing 7	25.27	34.35	-1.36	0.01911
B9EHJ3	B9EHJ3	Tight junction protein ZO-1	3495.70	4708.87	-1.35	0.04857
E9PX29	E9PX29	Spectrin beta chain	73.42	97.04	-1.32	0.00763
Q5SXC4	Q5SXC4	Vascular endothelial zinc finger 1	846.07	1110.48	-1.31	0.01479
Q9EPR2	PG12A	Group XIIA secretory phospholipase A2	57.63	75.54	-1.31	0.00205
Q9CWW7	CXXC1	CXXC-type zinc finger protein 1	32.58	42.54	-1.31	0.00275
Q9CRB2	NHP2	H/ACA ribonucleoprotein complex subunit 2	471.12	603.99	-1.28	0.04380
Q6PGH1	BUD31	Protein BUD31 homolog	901.97	1153.81	-1.28	0.01020
P28798	GRN	Progranulin	275.44	349.27	-1.27	0.00270
Q8R2Y9	SOSB1	SOSS complex subunit B1	258.92	328.24	-1.27	0.02765
A0A1L1STD6	A0A1L1STD6	InaF motif-containing 2	70.15	88.50	-1.26	0.02397
Q9CR59	G45IP	Growth arrest and DNA damage-inducible proteins-interacting protein 1	101.78	127.96	-1.26	0.02262
Q61072	ADAM9	Disintegrin and metalloproteinase domain-containing protein 9	110.56	138.73	-1.25	0.04969
Q99MI1	RB6I2	ELKS/Rab6-interacting/CAST family member 1	1080.81	1350.66	-1.25	0.01304
P62307	RUXF	Small nuclear ribonucleoprotein F	616.97	771.00	-1.25	0.02214
Q8R149	BUD13	BUD13 homolog	128.97	160.69	-1.25	0.04688
Q6ZQ58	LARP1	La-related protein 1	137.16	170.86	-1.25	0.03675

APP/PS1, $APP_{SWE}/PS1_{L166P}$ double transgenic mouse model of Alzheimer's disease (AD); APP/PS1:miR155cKO, $Cx3cr1^{CreERT2}$-$miR155^{fl/fl}$-APP/PS1 mice; FC, fold-change (FC>1.2 included); *P*<0.05

Table S7. Mass spectrometry data showing significantly up-regulated proteins in 4-month-old WT:miR155cKO compared to WT control group.

Accession	Symbol	Description	WT cKOmiR155 (Mean)	WT (Mean)	FC	p-value
Q8C6D4	BEND5	BEN domain-containing protein 5	1029.07	627.06	1.64	0.04189
Q8BTI9	PK3CB	Phosphatidylinositol 4,5-bisphosphate 3-kinase catalytic subunit beta isoform	1450.72	917.96	1.58	0.04940
Q8R2N0	TAP26	Thyroid transcription factor 1-associated protein 26	343.63	229.46	1.50	0.00576
Q8K4S1	PLCE1	1-phosphatidylinositol 4,5-bisphosphate phosphodiesterase epsilon-1	40.10	27.33	1.47	0.04304
Q9EPQ2	RPGR1	X-linked retinitis pigmentosa GTPase regulator-interacting protein 1	94.36	66.11	1.43	0.04276
Q66JT5	TPGS2	Tubulin polyglutamylase complex subunit 2	68.77	48.57	1.42	0.00104
G3UW87	G3UW87	####Predicted gene, 17455	362.86	260.30	1.39	0.02261
Q64518	AT2A3	Sarcoplasmic/endoplasmic reticulum calcium ATPase 3	242.86	174.55	1.39	0.02434
Q8BGZ2	F168A	Protein FAM168A	350.45	258.03	1.36	0.03473
P97318	DAB1	Disabled homolog 1	66.58	49.18	1.35	0.02041
Q9D903	EBP2	Probable rRNA-processing protein EBP2	501.79	373.16	1.34	0.02507
Q60953	PML	Protein PML	69.61	53.00	1.31	0.03010
Q3TZX8	NOL9	Polynucleotide 5'-hydroxyl-kinase NOL9	66.08	50.47	1.31	0.01111
Q60875	ARHG2	Rho guanine nucleotide exchange factor 2	54.18	41.41	1.31	0.02822
Q8R1F0	L10K	Leydig cell tumor 10 kDa protein homolog	1430.08	1096.18	1.30	0.01026
Q7TNM2	TRI46	Tripartite motif-containing protein 46	31.01	23.96	1.29	0.01015
Q8VIJ8	NPRL3	GATOR complex protein NPRL3	17.88	13.88	1.29	0.00150
O88622	PARG	Poly(ADP-ribose) glycohydrolase	111.35	87.25	1.28	0.00952
Q9D1B9	RM28	39S ribosomal protein L28, mitochondrial	151.54	118.86	1.27	0.00428
Q99MS3	MP17L	Mpv17-like protein	184.34	145.91	1.26	0.03608
A6PWY4	WDR76	WD repeat-containing protein 76	38.06	30.15	1.26	0.03620
Q9QZ47	TNNT3	Troponin T, fast skeletal muscle	265.43	211.10	1.26	0.01216
E9Q1U1	CC171	####Isoform 2 of Coiled-coil domain-containing protein 171	413.97	329.28	1.26	0.01324
Q5BL07	PEX1	Peroxisome biogenesis factor 1	82.85	65.94	1.26	0.03727
E9PX29	E9PX29	Spectrin beta chain	97.24	77.75	1.25	0.00262

Q9DCU6	RM04	39S ribosomal protein L4, mitochondrial	171.36	137.34	1.25	0.00424
Q8BWS5	GRIN3	G protein-regulated inducer of neurite outgrowth 3	210.07	168.73	1.24	0.02071
Q9R118	HTRA1	Serine protease HTRA1	57.22	46.14	1.24	0.00562
F2Z3U3	F2Z3U3	Ras association (RalGDS/AF-6) and pleckstrin homology domains 1	149.35	120.80	1.24	0.02219
Q99PJ1	PCD15	Protocadherin-15	73.63	59.62	1.23	0.04482
P97789	XRN1	5'-3' exoribonuclease 1	101.78	82.43	1.23	0.01737
Q8C3X8	LMF2	Lipase maturation factor 2	65.43	53.01	1.23	0.01938
B9EHJ3	B9EHJ3	Tight junction protein ZO-1	4876.67	3953.52	1.23	0.03428
Q68FM7	Q68FM7	Rho guanine nucleotide exchange factor (GEF) 11	398.36	323.85	1.23	0.00563
Q5SU73	COIL	Coilin	349.34	284.39	1.23	0.03945
Q8VC66	ADIP	Afadin- and alpha-actinin-binding protein	81.80	66.94	1.22	0.01354
Q8K194	SNR27	U4/U6.U5 small nuclear ribonucleoprotein 27 kDa protein	440.65	361.01	1.22	0.02481
A2AQ19	RTF1	RNA polymerase-associated protein RTF1 homolog	494.90	406.13	1.22	0.01815
Q923Z3	MTO1	Protein MTO1 homolog, mitochondrial	32.54	26.77	1.22	0.00307
Q8BHF7	PGPS1	CDP-diacylglycerol--glycerol-3-phosphate 3-phosphatidyltransferase, mitochondrial	147.91	121.89	1.21	0.01325
Q99J56	DERL1	Derlin-1	86.84	71.84	1.21	0.04780
Q3UQA7	SELH	Selenoprotein H	475.48	393.78	1.21	0.02780
G3X9N3	G3X9N3	PNMA-like 2	258.04	214.32	1.20	0.02339
P22682	CBL	E3 ubiquitin-protein ligase CBL	20.68	17.31	1.20	0.02113

WT,wild type mice; WT:miR155cKO, Cx3cr1^{CreERT2}-miR155$^{fl/fl}$-WT mice; FC, fold-change (FC>1.2 included); *P*<0.05

Table S8. Mass spectrometry data showing significantly down-regulated proteins in 4-month-old WT:miR155cKO compared to WT control group.

Accession	Symbol	Description	WT:miR155cKO (Mean)	WT (Mean)	FC	p-value
A0A0R4J0I1	A0A0R4J0I1	Serine protease inhibitor A3K	714.09	1059.25	-1.48	0.02849
Q8CH19	CS2IP	Casein kinase II subunit alpha'-interacting protein	318.75	470.98	-1.48	0.00029
Q99JW4	LIMS1	LIM and senescent cell antigen-like-containing domain protein 1	43.31	59.02	-1.36	0.02235
F6ZGI7	F6ZGI7	Molybdopterin synthase sulfur carrier subunit	57.01	75.14	-1.32	0.01053
Q9R0P5	DEST	Destrin	1723.22	2224.28	-1.29	0.02363
Q01768	NDKB	Nucleoside diphosphate kinase B	245.77	315.11	-1.28	0.04935
Q5NBX1	COBL	Protein cordon-bleu	47.68	59.89	-1.26	0.03302
Q9R0P9	UCHL1	Ubiquitin carboxyl-terminal hydrolase isozyme L1	3903.87	4867.96	-1.25	0.01349
Q921G6	LRCH4	Leucine-rich repeat and calponin homology domain-containing protein 4	16.07	20.00	-1.24	0.02973

WT,wild type mice; WT:miR155cKO, Cx3cr1^{CreERT2}-miR155$^{fl/fl}$-WT mice; FC, fold-change (FC>1.2 included); *P*<0.05
